# Neofunctionalization underlies the evolutionary origin of sclareol biosynthesis in the mint family

**DOI:** 10.1038/s41467-026-73637-5

**Published:** 2026-05-22

**Authors:** Fei Dong, Marion Verdenaud, Gabriele Adam, Feng-Quan Tan, Wissame Mouloud, Stephanie Drevensek, Melissa Hanique, Clement Pichot, Fabien Marcel, Francoise Gilard, Bertrand Gakière, Alexandra Launay-Avon, Etienne Delannoy, Benoit Join, Johannes Panten, Michel Dron, Abdelhafid Bendahmane, Adnane Boualem

**Affiliations:** 1 Université Paris Cité, CNRS, INRAE, Institute of Plant Sciences Paris-Saclay (IPS2), Gif-sur-Yvette, France; 2 Université Paris-Saclay, CNRS, INRAE, Université d’Evry, Institute of Plant Sciences Paris-Saclay (IPS2), Gif-sur-Yvette, France; 3 Global Innovation Scent & Care, Symrise AG, Holzminden, Germany

**Keywords:** Secondary metabolism, Comparative genomics, Natural product synthesis, Genetic variation

## Abstract

Plant specialized metabolites play essential ecological roles, yet the mechanisms underlying their diversification remain poorly understood. Here, we investigate the biosynthesis of sclareol, a potent antifungal diterpene produced by *Salvia sclarea* (clary sage). A complete telomere-to-telomere genome assembly of clary sage, compared with genomes of related Lamiaceae species that do not produce sclareol, reveals a recent tandem duplication of a class II diterpene synthase gene (*SsLPPS*). This duplicated enzyme acquires a specific catalytic activity, synthesizing labda-13-en-8-ol diphosphate (LPP), the direct precursor of sclareol. Structural modeling and site-directed mutagenesis identify key amino acid substitutions responsible for this neofunctionalization. Integrative genome, chromatin, and transcriptome analyses show that *SsLPPS* and additional diterpenoid biosynthetic genes are organized in a trichome-specific, co-regulated gene cluster. Together, our findings illustrate how enzyme innovation and regulatory rewiring can give rise to unique metabolic pathways and may inform future strategies for engineering valuable plant terpenoids.

## Abstract (web summary)

Within the mint family, clary sage (*Salvia sclarea*) is the only species that produces sclareol, a pathogen defense diterpene. Here, the authors report a chromosome level clary sage genome, a genus level pangenome consisting of seven assemblies, and the gene underlying sclareol biosynthesis.

## Introduction

Plants synthesize a remarkable diversity of specialized metabolites that are essential for interacting with their environment. These molecules play important ecological roles by protecting against herbivores and diseases, helping plants cope with abiotic stresses, influencing relationships within the rhizosphere and phyllosphere, and attracting pollinators and helpful microbes^1^. Although over 200,000 specialized metabolites have been identified in plants, the genetic and evolutionary processes underlying their diversification remain largely unresolved. Advances in high-throughput sequencing and comparative evolutionary genomics, across related species, offers a powerful approach to uncover the underlying mechanisms.

The Lamiaceae, or mint family, presents an exceptional model for exploring the evolution of specialized metabolism, owing to its remarkable diversity of bioactive compounds, including terpenoids, flavonoids, and phenolic acids^2–4^. These metabolites play essential roles in plant defense and ecological interactions, while also being highly valued for their medicinal, aromatic, and industrial uses. Among its members, *Salvia sclarea* (clary sage) is notable for producing sclareol, a labdane-type diterpenoid with dual functions as a potent antifungal defense compound and a commercially important precursor for ambergris substitutes in the fragrance industry^5^.

Sclareol biosynthesis begins with the universal diterpene precursor geranylgeranyl diphosphate (GGPP), produced via the plastidial MEP pathway. A class II diterpene synthase (diTPS) first converts GGPP into a labdane-type intermediate, typically copalyl diphosphate (CPP) or a closely related structure. This intermediate is then transformed by a class I diTPS into sclareol through cyclization and hydroxylation^6^. These enzymatic steps are tightly regulated and occur specifically in the glandular trichomes (GTs) of floral tissues, where sclareol accumulates^5^. This unique localization offers a powerful model for studying the evolution of tissue-specific metabolism and the genetic innovations that enable such specialization. While the enzymatic steps of sclareol biosynthesis have been previously described, the evolutionary origin, molecular mechanism, and regulatory integration of this pathway remain unresolved.

Here, we use sclareol biosynthesis as a model to dissect how gene duplication, structural divergence, and chromatin specialization jointly give rise to a distinct enzymatic function. We present a chromosome-level, telomere-to-telomere (T2T) assembly of the *S. sclarea* genome. Comparative genomic analyses with related diterpene-producing species reveal a species-specific tandem duplication of a class II diterpene synthase gene. Functional assays, protein structural modeling, and chromatin accessibility profiling further demonstrate that both enzyme neofunctionalization and changes in transcriptional regulation lead to sclareol biosynthesis in glandular trichomes (GTs). Together, our findings reveal evolutionary processes that give rise to specialized metabolic functions in plants.

## Results

### T2T genome assembly, annotation, and centromerearchitecture of *S. sclarea*

**Fig. 1:**
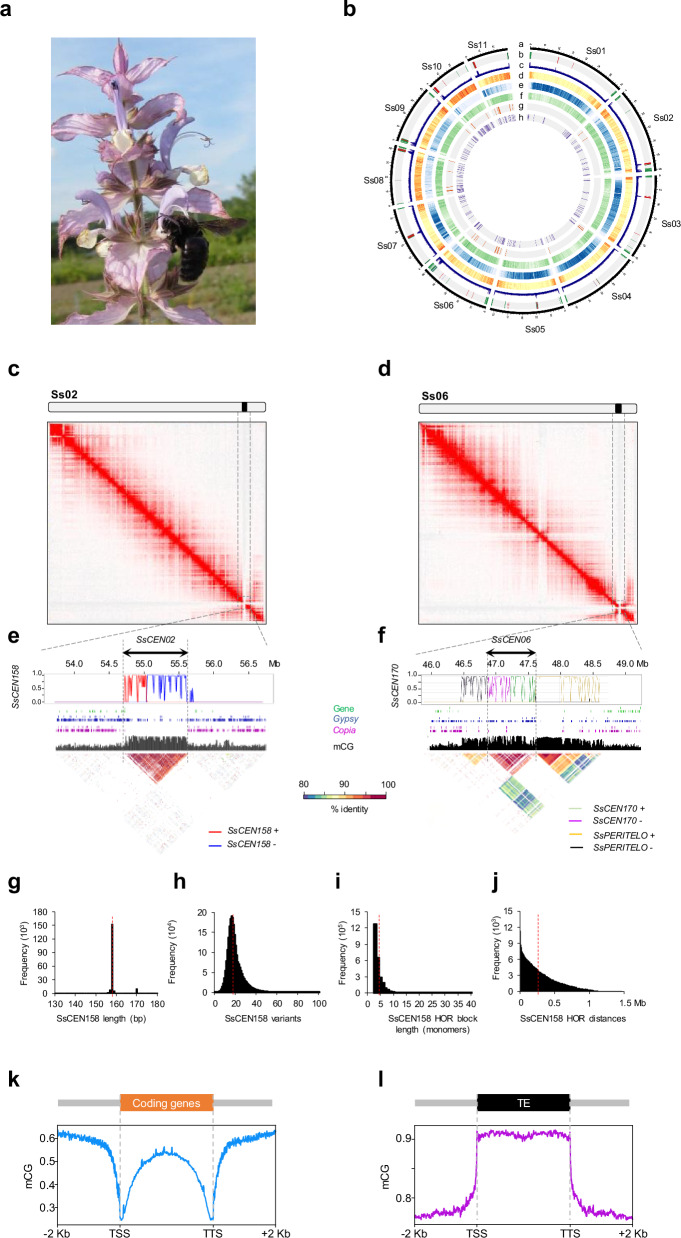
Genome assembly and centromere characterization of the telomere-to-telomere *S. sclarea* genome. **a** Morphology of the *S. sclarea* inflorescence. **b** Circos plot of the *S. sclarea* genome. Genomic tracks, shown in 100-kb bins, include: **a** chromosomes, **b** tandem repeats (centromeric in red; subtelomeric in green), **c** CG methylation, **d** gene density, **e** Class I transposable elements (TEs), **f** Class II TEs, **g** terpene synthase (TPS) genes, and **h** cytochrome P450 (CYP450) genes. **c**, **d** Hi-C contact maps of chromosomes Ss02 and Ss06. Full-genome contact maps are shown in Fig. S2. **e**, **f** Centromere composition and epigenetic features of chromosomes Ss02 and Ss06. Shown are the densities (per 10 kb) of centromeric tandem repeats *SsCEN158*, *SsCEN170*, and *SsPERITELO*; gene locations (green); *GYPSY* (dark blue) and *COPIA* (magenta) elements; CG methylation (black); and sequence similarity heatmaps. All eleven centromeres are shown in Fig. S3. **g–j** Distribution of centromeric repeat features: **g** monomer lengths of *SsCEN158*, **h** variant distances to the genome-wide *SsCEN158* consensus, **i** number of monomers per higher-order repeat (HOR), and **j** inter-HOR distances. Red dashed lines indicate mean values. **k**, **l** Average CG methylation levels across genic regions (**k**) and transposable elements (**l**), including 2 kb upstream and downstream of each feature. TSS transcription start site, TTS transcription termination site.

To generate T2T assembly of the *S. sclarea* genome, we developed a highly homozygous line and confirmed its diploid karyotype (2n = 2x = 22; Supplementary Fig. 1a, b). K-mer analysis estimated a genome size of 472.16 Mb with 0.131% heterozygosity (Supplementary Fig. 1c). We performed de novo hybrid assembly using 27.29 Gb of PacBio HiFi reads, 44.36 Gb of Oxford Nanopore long reads, and 94.94 Gb of Illumina short reads, corresponding to ~50, 82, and 175× coverage, respectively (Supplementary Table 1). The resulting 542.29 Mb assembly comprised 15 contigs with an N50 of 45.48 Mb (Table 1). Hi-C scaffolding further refined the assembly into 11 pseudochromosomes (Ss01–Ss11), with nine chromosomes assembled as single contigs (Fig. 1a, b, Supplementary Fig. 2, and Supplementary Table 2). This T2T assembly exhibits substantially higher contiguity and completeness than the *S. sclarea* genome reported by ref. ^7^, including full resolution of telomeric and centromeric regions and recovery of nearly twice as many non-TE gene models (Supplementary Table 3).

**Table 1:** Global statistics of *S. sclarea* genome assembly and annotation

Genomic feature	Statistics
Number of chromosome	11
Number of contig	15
Total length (bp)	542 293 756
GC content (%)	38.95
Number of telomeres	20
Number of centromeres	11
Number on non-TE gene models	32 992
Complete BUSCOs (%)	99.3
LAI	23

Centromeres in the *S. sclarea* genome were defined by integrating Hi-C interaction profiles with a genome-wide tandem repeat search using TRASH^8^. Centromeric regions corresponded to Hi-C “blank” zones enriched in satellite repeats, totaling 192,311 elements and spanning 10.9 Mb across the 11 chromosomes (Fig. 1c–f, Supplementary Fig. 3, and Supplementary Table 4). These regions were dominated by *a* ~ 158 bp satellite (CEN158), sharing over 98% identity with a FISH-validated marker^9^ (Fig. 1g and Supplementary Fig. 4). CEN158 repeats enabled the assignment of centromere positions (SsCEN01–SsCEN11) and formed strand-specific sequence blocks across most centromeres, except SsCEN10 (Supplementary Fig. 3). SsCEN06 harbored a distinct 170 bp satellite variant (CEN170), highlighting centromere-specific divergence (Fig. 1f). Each centromere maintained a private monomer library, with an average of 19 substitutions per 158 bp unit (Fig. 1h and Supplementary Fig. 5).

CEN158 satellites were organized into 2.7 million higher-order repeats (HORs), averaging 4.35 monomers and forming localized clusters within centromeres (Fig. 1i, j, Supplementary Fig. 6, and Supplementary Table 5). HOR arrays were frequently interrupted by LTR retrotransposons (LTR-RTs), particularly Gypsy-type elements, which accounted for 75% of centromeric sequence, double that of non-centromeric regions (Supplementary Fig. 7a, b). Insertion time analysis revealed that centromeric LTR-RTs were significantly younger than those elsewhere in the genome, suggesting recent and active turnover (Supplementary Fig. 7c).

The completeness of the assembly is underscored by the identification of 20 telomeres based on conserved telomeric repeats (TTTAGGG), confirming a true T2T assembly (Supplementary Fig. 1d and Supplementary Table 6), as well as the presence of two 5S rDNA loci on chromosomes Ss08 and Ss10 (Supplementary Table 7). The high assembly quality was supported by a BUSCO score of 99.3% and a Long Terminal Repeat Assembly Index (LAI) of 23, exceeding the “gold standard” threshold (>20) for plant genomes^10^ (Table 1 and Supplementary Table 8).

Gene annotation integrating ab initio prediction, homology-based, and transcriptomic evidence identified 32,992 protein-coding genes, with an average CDS length of 1,065.63 bp (Supplementary Table 9). Repetitive elements accounted for 61.77% of the genome, with LTR retrotransposons being the most abundant class at 43.74% (Supplementary Table 10). Insertion time analysis of 8020 intact LTR-RTs revealed recent bursts around 0.1 million years ago (Supplementary Fig. 8 and Supplementary Table 11).

Epigenetic profiling using long-read methylation data revealed canonical gene-body CG methylation, with depletion at transcription start and termination sites (Fig. 1k). Transposable elements exhibited high internal methylation, while centromeres showed elevated CG methylation relative to arms (Fig. 1e, f, l), suggesting a role in heterochromatin stability and centromere integrity. Together, these results provide a high-resolution view of the genomic and epigenetic landscape underlying the *S. sclarea* genome.

### *Salvia* gene-based pan-genome construction

**Fig. 2:**
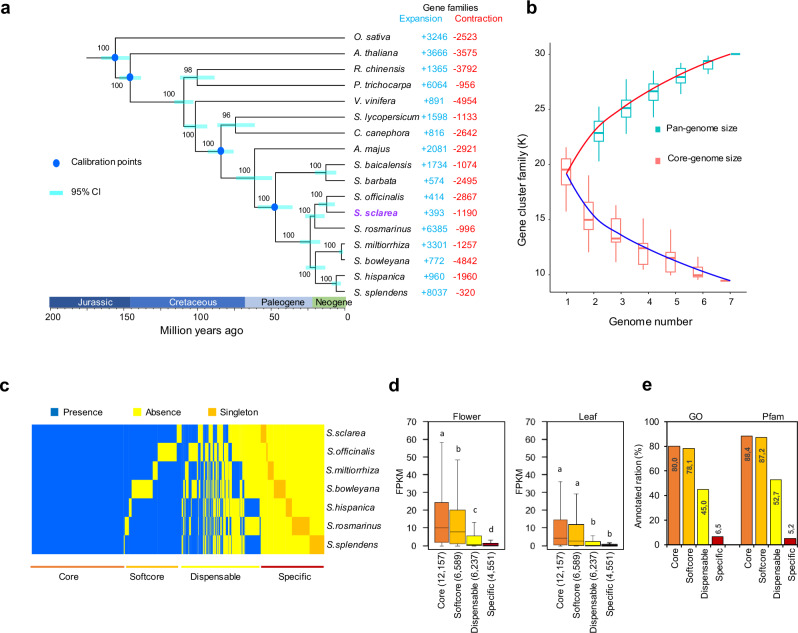
Pan- and core-genome analyses of *Salvia* species. **a** Phylogenetic tree of *S. sclarea* and 16 representative angiosperm species. Light blue bars represent the 95% confidence intervals for estimated divergence times. Numbers of gene family expansions (blue) and contractions (red) are indicated at each node. **b** Dynamics of gene family accumulation in the *Salvia* pan-genome and reduction in the core genome based on pairwise comparisons among 7 *Salvia* genomes. **c** Heatmap displaying presence/absence variation (PAV) for pan-gene families across the 7 *Salvia* genomes. **d** Expression levels of pan and core genes in *S. sclarea* flower and leaf transcriptomes. *n* =  gene counts in each category (shown in parenthesis). **e** GO and Pfam annotation profiles for pan and core genes. Boxplots represent median (center lines), interquartile range (boxes), and 1.5× interquartile range (whiskers). Statistical analysis was performed using a one-way ANOVA, followed by two-sided least significant difference (LSD) post hoc tests. Multiple comparisons were adjusted using the Bonferroni correction. Significant pairwise differences are indicated by compact letter display (CLD), where different letters denote statistically significant differences (*P* < 0.05).

To elucidate the process of *S. sclarea* genome evolution, we analyzed the genetic relationship of 17 representative angiosperm species, including eight Lamiaceae members. We dated the split of the Lamiaceae from the rest of the Lamiales lineage around 61 million years ago (MYA), with *Scutellaria* and *Salvia* genera splitting around 47 MYA (Fig. 2a). *Salvia* genus forms a monophyletic group that diversified later in 3 clades, the Mediterranean (*S. officinalis*, *S. sclarea*, and *S. rosmarinus*), the America (*S. splendens and S. hispanica*), and the East Asia (*S. miltiorrhiza and S. bowleyana*), highlighting long-distance dispersal as a likely driver of speciation (Fig. 2a).

Comparative genome annotation analysis identified 36,737 gene families, including 6013 shared across all 17 species, 8480 shared by all *Salvia* species, and 904 specifics to *S. sclarea* (Supplementary Fig. 9a). A *Salvia* pan-genome constructed from seven assemblies identified 30,316 gene clusters encompassing 305,116 genes (Fig. 2b). Core clusters (shared by all genomes) accounted for 31.2% of clusters and 44.1% of genes. The remainder included softcore (19.6%), dispensable (27.2%), and species-specific (21.9%) clusters, indicating high intra-genus gene content variability (Fig. 2c and Supplementary Fig. 9b). Core genes exhibited broader expression across tissues and higher functional annotation, whereas species-specific genes showed low or highly restricted expression across the sampled tissues, often detectable only under particular tissues or conditions, reflecting a pattern that is generic across plant pan-genomes (Fig. 2d, e). In *S. sclarea*, these conserved and broadly expressed core and soft-core families provide a stable genomic background from which lineage-specific expansions in specialized metabolism, particularly TPS and CYP450 families, have diversified. CYP450 enzymes catalyze a wide range of oxidative modifications that decorate terpene scaffolds and generate much of the structural diversity observed in plant specialized metabolites^11,12^.

Among the 8480 gene families shared across all *Salvia* species, 1583 exhibited rapid evolutionary changes in the *S. sclarea* lineage, including 393 expansions and 1190 contractions (Fig. 2a). GO enrichment of the expanded families highlighted secondary metabolism (*p* < 0.01) (Supplementary Fig. 9c, d). Of the 65 expanded genes annotated with this function, 57% corresponded to cytochrome P450s (CYP450s), enzymes broadly involved in specialized metabolism such as terpenoids, flavonoids, alkaloids, and phenylpropanoids^11,12^. The *S. sclarea* genome encodes 402 CYP450 genes grouped into nine clans (Supplementary Data 1). Phylogenetic analysis of 313 full-length CYP450s (>300 aa) showed that the CYP71 clan comprises 17 families and 185 genes, whereas the remaining eight clans encompass 31 families and 128 genes (Supplementary Fig. 10a and Supplementary Data 1). CYP71 and CYP76 families, central to Lamiaceae diterpenoid diversification^13,14^, are highly represented and frequently organized in genomic clusters, notably on chromosome 8, where cluster 8.1 contains 11 CYP76 and 9 CYP71 genes in close proximity (Supplementary Fig. 10b–d).

To place sclareol biosynthesis within this broader evolutionary framework, we also examined the diversification of other specialized-metabolism gene families in *S. sclarea*, including terpene synthases (TPSs) (Supplementary Data 2). Although CYP450s are not involved in the terminal steps of sclareol formation, their lineage-specific expansions, together with dynamic changes in TPS families, underscore the high genomic plasticity of *Salvia* specialized metabolism. These patterns highlight the diverse trajectories diterpenoid pathways have taken across Lamiaceae and provide the comparative context necessary to understand the lineage-specific emergence of sclareol biosynthesis in *S. sclarea*.

### LPP synthase originated from a recent CPS gene duplication and neofunctionalization

**Fig. 3:**
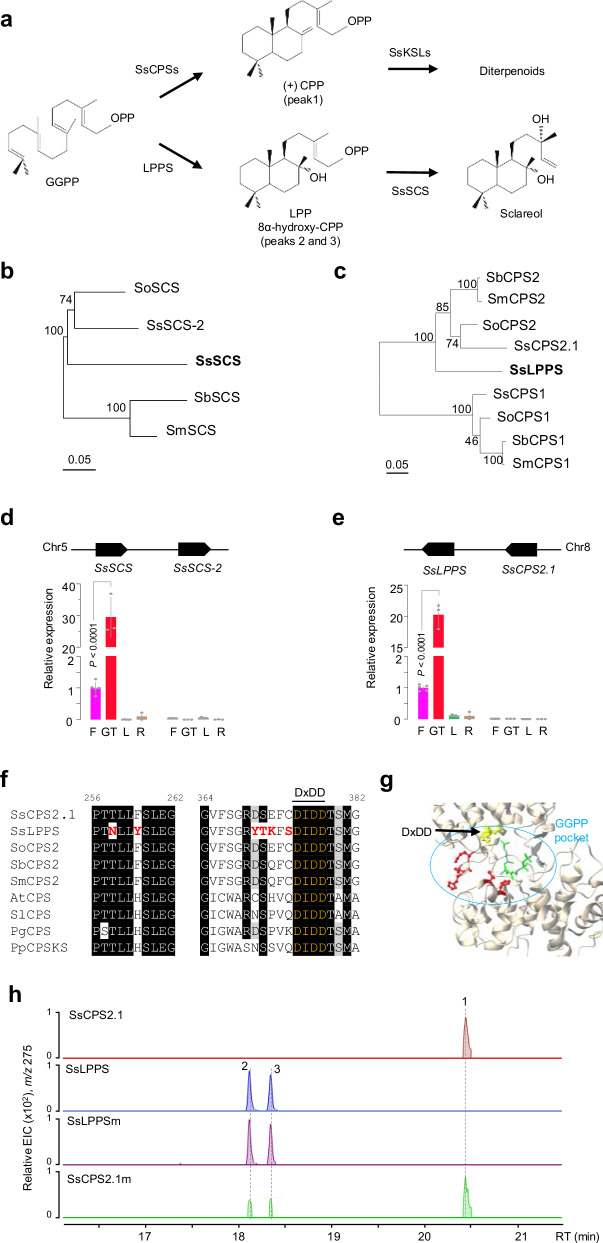
Functional analysis of SsCPS2.1 and SsLPPS. **a** Proposed biosynthetic pathway of sclareol and related diterpenes in *S. sclarea*. **b**, **c** Phylogenetic trees of class I diTPSs (SCS-like, **b**) and class II diTPSs (CPS-like, **c**) from *S. sclarea*, compared with homologs from *S. officinalis* (So), *S. miltiorrhiza* (Sm), and *S. bowleyana* (Sb). **d**, **e** qRT–PCR analysis of *SsLPPS*, *SsCPS2.1*, *SsSCS*, and *SsSCS-2* expression in flower buds (F), glandular trichomes (GT), leaves (L), and roots (R). Data are presented as mean ± s.d. (*n* = 3 biological replicates for GT and R; *n* = 5 biological replicates for F and L). Statistical significance was assessed by two-tailed Student’s *t*-test. **f** Protein alignment of SsLPPS with CPS2 homologs from *Salvia* spp. (So, Sb, Sm), *A. thaliana* (At), *S. lycopersicum* (Sl), *P. glauca* (Pg), and *P. patens* (Pp), highlighting key active site residues involved in product specificity. **g** Structural model of SsCPS2.1 showing the conserved DXDD motif (yellow), the substrate GGPP (green), and key residues (T258, F261, D370, S371, E372, and C374S in red) targeted by site-directed mutagenesis. **h** In vitro enzymatic activity of wild-type and mutant SsCPS2.1 and SsLPPS using GGPP as substrate. Shown are extracted ion chromatograms (EIC, *m/z* 275) for SsCPS2.1 (red), SsLPPS (blue), SsLPPSm (purple), and SsCPS2.1 m (green). Peak 1: (+)-copalol (dephosphorylated CPP); peaks 2 and 3: (13R)- and (13S)-manoyl oxides (LPP). Corresponding mass spectra are shown in Supplementary Fig. 14. Source data are provided as a Source Data file.

Comparative genomic analysis underscored the diversification of genes related to secondary metabolism during the evolution of *Salvia* species. Since *S. sclarea* is the main natural source of sclareol, we focused our analysis on the diversification of the genes responsible for its production. Sclareol is biosynthesized via two enzymatic steps catalyzed by monofunctional diterpene synthases (diTPSs): a class II diTPS first converts geranylgeranyl diphosphate (GGPP) to labda-13-en-8-ol diphosphate (LPP), followed by a class I diTPS that produces sclareol^6^ (Fig. 3a). A genome-wide search identified 52 class I and 5 class II TPSs containing the characteristic DDXXD and DXDD motifs^15^, respectively, classified into six families: TPSa (19), TPSb (25), TPSc (5), TPSe (6), TPSf (1), and TPSg (1) (Supplementary Fig. 11 and Supplementary Data 2).

Among the seven class I diTPSs, four encode kaurene synthase-like (KSL) enzymes, including the tandem-duplicated sclareol synthases SsSCS and SsSCS-2 (Fig. 3b, j). Five class II diTPSs were identified, including copalyl diphosphate synthase 1 (SsCPS1), SsCPS2.1, and its tandem duplicate SsLPPS, which encodes labda-13-en-8-ol diphosphate synthase^6^, the enzyme producing LPP, the immediate precursor of sclareol (Fig. 3c, i). Phylogenetic and synteny analyses indicated that SsLPPS arose through tandem duplication and neofunctionalization of SsCPS2.1 after the divergence of *S. sclarea* and *S. officinalis* (~13 MYA).

To determine whether these tandem duplications were accompanied by changes in gene regulation, we examined their expression across tissues. Expression profiling showed that *SsSCS* and *SsLPPS* are strongly expressed in GTs, consistent with the site of sclareol accumulation (Fig. 3d, e), whereas *SsSCS-2* and *SsCPS2.1* transcripts were undetectable in the tissues examined. Together, these results indicate that the emergence of sclareol biosynthesis in *S. sclarea* involved both gene duplication and the acquisition of trichome-specific gene regulation.

To investigate the molecular basis of catalytic divergence, we constructed homology models of *SsCPS2.1* and *SsLPPS* using the crystal structure of *Arabidopsis* CPP synthase and AlphaFold structural predictions^16,17^. Although both enzymes share conserved catalytic cores, molecular docking of GGPP revealed distinct active-site configurations. In particular, residues T258, F261, D370, S371, E372, and C374 were positioned closest to the substrate, influencing the polarity and steric constraints of the active-site pocket (Fig. 3f, g and Supplementary Figs. 12 and 13). Notably, these residues are conserved in CPP-producing CPSs across angiosperms, gymnosperms, and bryophytes, reflecting over 450 million years of evolutionary conservation (Fig. 3h). In vitro assays confirmed functional divergence between SsCPS2.1 and SsLPPS. SsCPS2.1 catalyzed the formation of copalol, the dephosphorylated form of CPP (peak 1), whereas SsLPPS produced a diastereomeric mixture of (13S)- and (13R)-manoyl oxide, representing dephosphorylated LPP^18^ (peaks 2 and 3) (Fig. 3h and Supplementary Fig. 14). To assess the impact of the polymorphic active-site residues on enzyme specificity, we modified SsCPS2.1 to mimic the active site of SsLPPS by introducing six substitutions: T258N, F261Y, D370Y, S371T, E372K, and C374S. The resulting variant, SsCPS2.1 m, gained the ability to produce both (13S)- and (13R)-manoyl oxide, in addition to copalol (Fig. 3h). In contrast, the reciprocal mutation, restoring the corresponding SsCPS2.1 residues into the SsLPPS active site, did not recover CPP synthase activity (Fig. 3h). The resulting SsLPPSm mutant variant retained LPP activity, indicating that additional structural changes likely disrupt CPS functionality. Three-dimensional structural analysis of polymorphic amino acids between SsLPPS and SsCPS2.1 enzymes revealed a mutation hotspot near the active site, suggesting that these substitutions likely have disrupted CPS function during the neofunctionalization of SsLPPS (Supplementary Figs. 12 and 13). Together, these results demonstrate that a small number of amino acid substitutions were sufficient to causally reprogram enzyme specificity, providing direct molecular evidence for enzymatic neofunctionalization following gene duplication.

### Diterpene biosynthetic genes are arranged in co-expressed gene clusters

**Fig. 4:**
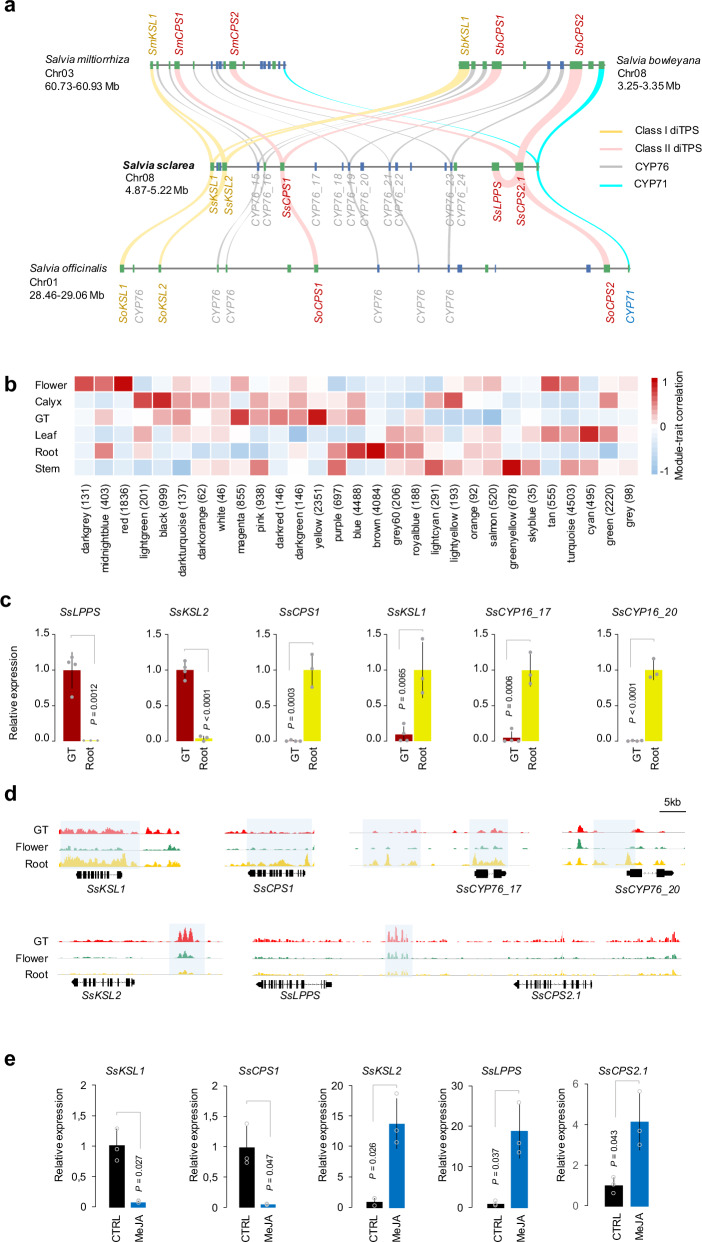
Evolution of the *Salvia* metabolic gene cluster. **a** Genomic organization and microsyntenic relationships of the diterpene biosynthetic gene cluster in *Salvia* species. Syntenic relationships between species are illustrated by colored curves. **b** WGCNA modules associated with GTs and roots. Modules are indicated by their standard color-based names (e.g., midnightblue), with the number of genes in each module shown in parentheses. **c** Relative expression levels of the BGC genes in GT and roots. Data are presented as mean ± s.d. (*n* = 4 biological replicates for GT; *n* = 3 biological replicates for Roots) (**d**) ATAC-seq chromatin accessibility profiles at the BGC loci in GTs, flowers, and roots. Open chromatin regions are shaded in light blue. **e** qRT–PCR analysis of *SsKSL1*, *SsCPS1*, *SsKSL2*, *SsLPPS*, and *SsCPS2.1* following methyl jasmonate (MeJA) treatment. Data are presented as mean ± s.d. (*n* = 3 biological replicates). *P* values were calculated using a two-tailed Student’s *t*-test. Source data are provided as a Source Data file.

In *S. sclarea*, genes associated with diterpene biosynthesis are spatially clustered within *a* ~ 350 kb biosynthetic gene cluster (BGC) located on chromosome 8 (Fig. 4a). This region encompasses five diterpene synthase (diTPS) genes and eleven cytochrome P450 monooxygenases (CYP450s), predominantly from the CYP76 family, all exhibiting coordinated expression. Comparative synteny analysis across four *Salvia* species (*S. sclarea, S. officinalis, S. miltiorrhiza*, and *S. bowleyana*) indicates that the core structure of this BGC is conserved, suggesting its establishment predates the diversification of the genus. Despite this conservation, variation in gene copy number and the presence of non-syntenic diTPS and CYP450 genes suggest lineage-specific functional divergence. Notably, the BGCs in *S. sclarea* and *S. officinalis* are expanded relative to those in their East Asian counterparts, harboring additional *KSL* and *CYP76* genes, likely the result of tandem duplications that occurred following speciation (Fig. 4a).

To investigate whether the sclareol pathway genes exhibit coordinated regulation, we examined the transcriptional organization and chromatin accessibility of the diterpene BGC across tissues. Weighted gene co-expression network analysis (WGCNA) using transcriptomes from roots, stems, leaves, flowers, calyces, and GTs resolved the cluster into two transcriptionally coherent modules (Fig. 4b and Supplementary Fig. 15). The GT-enriched module (magenta) contained *SsLPPS* and *SsKSL2*, consistent with their roles in sclareol biosynthesis. The root-enriched module (blue) contained *SsCPS1*, *SsKSL1*, and multiple *CYP76* genes, matching the production of abietane-type diterpenoids in roots. Tissue-specific expression patterns were validated by qRT–PCR (Fig. 4c).

To determine whether these transcriptional differences are associated with changes in chromatin state, we performed ATAC-seq on GTs, flowers, and roots. Chromatin-accessibility profiling reveals that promoters of GT-expressed genes exhibit elevated accessibility in trichomes, whereas promoters of root-expressed genes show increased accessibility in root nuclei (Fig. 4d). Together, these results indicate that the diterpene BGC is partitioned into two physiologically distinct regulatory modules supported by tissue-specific chromatin accessibility, linking enzyme neofunctionalization with the coordinated acquisition of trichome-specific gene regulation.

To further assess whether the *S. sclarea* diterpene BGC functions as a coordinated transcriptional unit, we investigated its responsiveness to hormonal cues. Methyl jasmonate (MeJA), a well-characterized phytohormone involved in plant defense and the activation of specialized metabolism^19^, was used to probe whether the identified biosynthetic gene cluster is not only spatially but also hormonally coordinated. Following MeJA treatment, we observed a striking transcriptional reprogramming of the cluster. Genes associated with the root-specific module were significantly downregulated, whereas those of the GT-specific module, including *SsLPPS* and *SsKSL2*, showed strong induction (Fig. 4e). This hormonal response was accompanied by the upregulation of well-known MeJA-responsive transcriptional repressors belonging to the JAZ (Jasmonate ZIM-domain) family^20^ (Supplementary Fig. 16a), confirming activation of the jasmonate signaling pathway and reinforcing the link between jasmonate signaling and specialized metabolism.

To determine whether this transcriptional response is associated with changes in chromatin accessibility, we performed targeted ATAC-qPCR assays at promoters of key genes within the diterpene BGC. These analyses revealed a significant increase in chromatin accessibility at the promoters of *SsLPPS* and *SsKSL2* following MeJA treatment (Supplementary Fig. 16b), indicating that jasmonate signaling is accompanied by promoter-level chromatin remodeling at core genes of the sclareol biosynthetic pathway.

The contrasting hormonal responsiveness of the two modules demonstrates that the diterpene BGC exhibits coordinated transcriptional behavior under a defense-related stimulus, consistent with its functional integration and tissue-specific specialization. Together, these observations support the view that evolutionary assembly of the cluster involved not only gene duplication and neofunctionalization, but also the emergence of distinct regulatory modules aligned with GT and root functions.

## Discussion

Plants continually innovate chemically to survive in diverse ecological niches^21,22^, yet the genomic routes to this diversification are only beginning to be resolved. By integrating a telomere-to-telomere genome assembly with structural, biochemical, and chromatin analyses, we uncover the evolutionary origin, structural innovation, and regulatory coordination that gave rise to sclareol biosynthesis, a lineage-specific trait that distinguishes *S. sclarea* from other *Salvia* species. Our results show that gene duplication, neofunctionalization, and cis-regulatory divergence acted together to produce a specific diterpenoid biosynthetic capability over a relatively short evolutionary timescale.

Although earlier studies characterized enzymes capable of producing intermediates in sclareol biosynthesis^6^, they did not address how these activities evolved or identify the genetic changes responsible for their emergence. Our work fills this gap by uncovering the evolutionary mechanism that gave rise to the pathway: a recent tandem duplication followed by neofunctionalization of a class II diterpene synthase. By integrating comparative genomics, structural modeling, and targeted mutagenesis, we show that only a small number of amino acid substitutions reconfigured the active site of the neofunctionalized enzyme SsLPPS to produce labda-13-en-8-ol diphosphate (LPP), the immediate precursor of sclareol. This establishes a direct causal link between specific sequence changes and the acquisition of a lineage-specific catalytic function, an insight that aligns with broader principles of terpene synthase evolution yet is demonstrated here with exceptional mechanistic resolution.

Our analyses further reveal that the neofunctionalized diterpene synthase and its partner enzymes are embedded within a chromatin-regulated metabolic gene cluster that is both tissue-specific and hormonally responsive. The cluster organizes into two distinct transcriptional modules: a GT module responsible for sclareol biosynthesis and a root-specific module associated with abietane-type diterpenoids. Co-expression network reconstruction (WGCNA) and chromatin-accessibility profiling show that these modules are supported by distinct cis-regulatory landscapes. This inverse regulatory switch may concentrate target metabolite production where it is most needed while limiting metabolic cost elsewhere, mirroring the modular gene clusters seen in microbes and documented in a few plants^23–27^. We also demonstrate that genes in the trichome module respond coherently to jasmonate signaling, consistent with their functional integration in a defense-related pathway. Together, these findings support a regulatory architecture in which tissue specialization and hormonal responsiveness contribute to the emergence and maintenance of a biosynthetic innovation.

Beyond illuminating sclareol biosynthesis, our phylogenomic and pan-genome analyses highlight the dynamic evolution of specialized-metabolism gene families across *Salvia*. Lineage-specific expansions of terpene synthases and cytochrome P450s illustrate how genomic plasticity provides the substrate for metabolic diversification throughout the Lamiaceae. These patterns provide an evolutionary framework for interpreting how distinct diterpenoid pathways, including those unrelated to sclareol, arose across the genus. Thus, the mechanistic insights uncovered here extend beyond *S. sclarea*, offering a comparative context for understanding diterpenoid evolution at the family level.

Together, our findings provide a high-resolution case study of how specialized metabolic traits originate in plants. By combining T2T genomics, enzyme structural biology, and integrative regulatory analysis, we reveal how duplication, neofunctionalization, and gene-cluster organization converged to create a lineage-specific diterpenoid. This work establishes *S. sclarea* as a tractable model for studying metabolic evolution in the Lamiaceae and offers a conceptual framework applicable to gene-cluster formation and specialized-metabolism innovation across plants. The mechanistic principles uncovered here, minimal catalytic rewiring, modular regulatory architecture, and tissue-specific deployment, are likely to be broadly relevant for both evolutionary biology and future metabolic engineering efforts.

## Methods

### Plant material and cytogenetics

*S. sclarea* seeds from the “Vatican White” population were obtained from Jelitto (Germany). Chromosome number was determined through standard cytogenetic techniques. Root tips were pretreated with 0.05% colchicine for 1 h at room temperature and fixed in absolute ethanol and glacial acetic acid (3:1) for at least one day. Root tips were then dissociated in 1 M HCl for 10 min in a 60 °C water bath, and stained in 1% orcein in 45% acetic acid for about 30 min and squashed for microscopic observation.

### Short- and long-read sequencing

Genomic DNA was extracted from leaf using the DNeasy Plant kit (Qiagen) according to the manufacturer’s instructions. DNA degradation and contamination were monitored on 1% agarose gels. DNA concentration was measured using a Qubit DNA Assay Kit in Qubit 2.0 Fluorometer (Life Technologies, CA). Sequencing libraries were generated using a NEB Next Ultra DNA Library Prep Kit for Illumina (NEB) following the manufacturer’s instructions and sequenced on an Illumina NovaSeq plat-form to generate 150 bp paired-end reads.

High molecular weight DNA was extracted from approximately 10 g of young *S. sclarea* leaves using a nuclei isolation and CTAB-based purification procedure optimized to preserve long DNA fragments^28^. DNA quality and integrity were assessed prior to library preparation and sequencing on the PacBio Sequel II (SMRT) and Oxford Nanopore PromethION (ONT) platforms. The sequencing was performed at Biomarker Technologies GmbH (Munster, Germany).

### Hi-C library construction and sequencing

Hi-C libraries were prepared from crosslinked nuclei using a chromosome conformation capture protocol adapted for plant tissues^29^. Chromatin was digested with DpnII, proximity-ligated, and processed for library construction using the NEBNext Ultra II DNA library preparation kit. Libraries were amplified by PCR (9 cycles), purified using SPRI beads (Beckman Coulter), and quality-controlled using an Agilent 2100 Bioanalyzer (Agilent). Sequencing was performed at the INRAE EPITRANS platform (Orsay, France) using paired-end 2 × 75 bp reads.

### ATAC-seq and ATAC-qPCR analysis

ATAC-seq was performed on nuclei isolated from approximately 10 g of young leaf tissue. Purified nuclei were subjected to Tn5 transposase-mediated chromatin tagmentation, followed by library preparation using the NEBNext Ultra II DNA library preparation kit (New England Biolabs). Libraries were purified using AMPure beads (E6220, New England Biolabs), quality-checked using an Agilent 2100 Bioanalyzer (Agilent, LabChip Caliper), and sequenced at the INRAE EPITRANS platform (Orsay, France).

Targeted ATAC-qPCR assays were performed on nuclei isolated from control and MeJA-treated *S. sclarea* tissues^30^. The resulting tagmented DNA was subjected to limited PCR amplification to generate ATAC libraries. Chromatin accessibility at specific loci was then quantified by qPCR using locus-specific primers designed within promoter regions of *SsLPPS* and *SsKSL2*. qPCR reactions were performed using MESA GREEN qPCR MasterMix Plus (Eurogentec) in a CFX384 Real-Time PCR System (Bio-Rad) with the following cycling conditions: 95 °C for 5 min followed by 40 cycles of 95 °C for 15 s and 60 °C for 1 min. Accessibility values were normalized to a closed intronic genomic region lacking detectable ATAC-seq signal, which served as a negative accessibility control. Primer sequences used for ATAC-qPCR are listed in Supplementary Data 3.

### RNA sequencing

Total RNA was extracted from flowers, glandular trichomes, leaves, stems, and roots using TRIzol reagent (Sigma-Aldrich), followed by purification with the EZNA RNA Purification Kit (Omega Bio-tek). RNA quantity and integrity were assessed using a Qubit® 3.0 Fluorometer (Thermo Fisher Scientific) and an Agilent 2100 Bioanalyzer (Agilent Technologies). Sequencing libraries (300 bp insert size) were prepared using the TruSeq Sample Preparation Kit (Illumina) and sequenced at the INRAE EPITRANS platform (Orsay, France).

For the MeJA treatment, *S. sclarea* plantlets were sprayed with either 1 mM methyl jasmonate (MeJA) containing 0.1% (v/v) Tween-20 or a control solution of 0.1% Tween-20 alone, using a total application volume of 50 mL per plant. Plants were covered with plastic bags immediately after spraying, and young leaves of comparable developmental stage were collected 6 h post-treatment. Total RNA from control and treated samples was extracted and purified as described above.

### Weighted gene co-expression network analysis (WGCNA)

To identify groups of genes exhibiting similar expression patterns across tissues in our transcriptome dataset, we constructed a gene co-expression network using the WGCNA package in R (v1.73). A one-step network construction approach was implemented using the blockwiseModules function with the following parameters: soft-thresholding power = 9, networkType = “signed hybrid”, maxBlockSize = 4000, minModuleSize = 30, reassignThreshold = 0.000006, mergeCutHeight = 0.35, and deepSplit = 2. This analysis identified 29 co-expression modules, each representing a set of genes with positively correlated expression profiles across the sampled tissues.

### Genome survey

PacBio HiFi reads were used to evaluate the genome size and heterozygosity rate of the *S. sclarea* genome by k-mer analysis; k-mers were counted by Jellyfish (www.genome.umd.edu/ jellyfish.html) with the k-mer size of 21. GenomeScope (https://github.com/schatzlab/genomescope) uses the k-mer count distribution to infer the global properties of *S. sclarea* genome.

### Genome assembly and quality control

PacBio HiFi reads were assembled using HiFiasm (v0.19.5) with default parameters. Leveraging the accuracy of HiFi long reads and the high continuity of the assembly, we directly performed scaffolding with the help of Hi-C seq data using HiCPro pipeline^31^ and YaHS software^32^. Considering the high continuity and integrity of the HiFi-based genome, we selected it as the genome backbone and filled the remaining gaps using the ONT reads. The telomere regions were identified by searching for the 7-bp telomeric repeat unit (CCCTAAA/TTTAGGG) using tidk software^33^. Genome completeness and quality were further assessed using Benchmarking Universal Single- Copy Orthologs, BUSCO (v.5.4.4, viridiplantae_odb10 dataset) with default parameters^34^.

### Annotation of protein-coding genes and repeat sequences

We integrated sequence homology, ab initio prediction, and transcriptomic approaches to build consensus gene models.

To annotate transposons and repeat sequences, we used External Tandem Repeat Annotation (EDTA) software^35^. To identify intact (retro)transposons and estimate insertion times, we used the LTR-retriever software^36^. Assuming the same mutation rate, the divergence was estimated between the left and right ends of the LTR sequence of each intact element.

For the ab initio approach, gene models were predicted with a fully automated and parallelized pipeline, egn-ep, that carries out probabilistic sequence model training, genome masking, transcript and protein alignment computation and integrative gene modeling in EuGene software^37^ (http://eugene.toulouse.inra.fr/). The expression data-driven approach was based on datasets that included short reads (Illumina) and long reads (Nanopore) RNAseq data. The RNA-seq datasets were derived from 5 tissues listed in Supplementary Table 1. Finally, gene models from these three methods were integrated into a non-redundant set of high-confidence gene models.

The functions of protein-coding genes were identified by mapping sequences against TrEMBL^38^ and TAIR^39^ databases using BLASTP (*E*-value 1 × 10^−5^) and integrating results of different tools (eggNOG, InterProScan, and PlantTFCat)^40–42^.

To predict centromeric regions, we integrated the Hi-C interaction heat map, the density distribution of satellite DNA repeats, and the density distribution of LTR-RTs. Tandem repeats with monomer lengths ranging between 80 and 2000 bp were identified using Tandem Repeats Finder (TRF), Tandem Repeat Annotation and Structural Hierarchy (TRASH), and EDTA tools^35,43,44^. The StainedGlass package was used to generate sequence identity heat maps^45^. The quality of repeat sequences was assessed using the LTR Assembly Index (LAI) from LTR retriever tool.

### Whole-genome CpG methylation methylation analysis

PacBio primrose v.1.3.0 (now Jasmine) was used to call CpG methylation from HiFi reads. Methylation probabilities encoded in the BAM tags ML and MM were parsed to continuous values for single-molecule methylation prediction. Per-CpG methylation was estimated using the tools available at github.com/PacificBiosciences/pb-CpG-tools.

### Phylogenetic analysis and divergence time estimation

To investigate the evolution of the *S. sclarea* genome, we selected 17 representative plant genomes, including species of Poaceae (*Oryza sativa*), Eurosid (*Vitis vinifera*, *Rosa chinensis*, *Arabidopsis thaliana*, *Populus trichocarpa*), Asterales (*Coffea canephora, Solanum lycopersicum*), Lamiales (*Antirrhinum majus*), and 8 Lamiaceae species (*Salvia splendens*, *Salvia hispanica, Salvia miltiorrhiza*, *Salvia bowleyana, Salvia rosmarinus, Salvia officinalis, Scutellaria baicalensis, and Scutellaria barbata*).

Orthologous groups (orthogroups) were inferred by Orthofinder (V2.4.0)^46^. The protein sequences from each genome were filtered by keeping the longest isoform and were fed to OrthoFinder with the following parameters: multiple sequence alignment MAFFT, sequence search BLASTP, and tree inference FastTree.

The neutral evolutionary rate and species divergence times were estimated using the MCMCTREE from PALM (v4.9j) package^47^. Changes in gene family size were analyzed by CAFE v5.0 (github.com/ hahnlab/CAFE) to calculate the expansions and contractions of gene families for each species. Genes from expanded gene families in *S. sclarea* were extracted to carry out the Gene Ontology annotation using ClusterProfiler (v2.5.2)^48^.

Chromosomal synteny between the genomes was analysed using MCScanX with default parameters^49^, and microsynteny visualization was drawn using JCVI program^50^.

### Gene-based pan-genome construction

A total of 7 genomes were used to construct a pan-genome of the *Salvia* genus. Core and variable gene families were counted based on gene family using Orthofinder (V2.4.0)^46^. We divided gene families into core, softcore, dispensable, and species-specific family genes, which were present in 7, 6, 2–5, and one genome, respectively. Protein-coding genes in each genome were iteratively added to the rest of the genomes, and this operation was repeated until all genes from 12 assemblies had been added to the pan-genome. A nonlinear regression model was used to fit the pan-gene saturation curve using the NLS function of R package (v 4.0.3).

### Identification of diTPS and CYP450s genes and gene clusters

For the identification and classification of diTPS and CYP450 genes across the *S. sclarea* genome, we applied a joint methodology including Pfam searching, homologous alignment, and syntenic analysis. For diTPS, two Pfam domains, PF01397 (terpene synthase, N-terminal domain) and PF03936 (terpene synthase family, metal binding domain), were used to search against the whole genome by HMMER v3.3 (hmmer.org). All the characterized class I and class II diTPS genes in Lamiaceae plants, which were retrieved from NCBI, were searched against the *S. sclarea* protein sequences using Blastp with threshold *e*-value < 10e-5 and coverage >50%.

To identify CYP450 genes, Pfam domains, PF00067 was used to search against the whole *S. sclarea* genome. The classification of the 402 CYP450 genes was executed by alignment with the CYP450 database^51^ using standard sequence similarity cut-offs. CYP450s were divided into clans^52^, and phylogenetic analysis of CYP450 genes was performed. Gene clusters were identified by in silico analysis on the basis of the following criteria: (1) the distance between two adjacent CYP450 genes in one group should be less than 1 Mb, and (2) one group should contain at least four CYP450 genes.

Neighbour-joining phylogenetic trees and multiple sequence alignments of all the identified proteins were performed using the MEGAX package^53^.

### Identification of enzymes in the terpene biosynthesis pathway

Gene coding enzymes in the MVA and MEP pathways of terpene biosynthesis were identified by an incorporated approach. The Pfam domains associated with enzymes involved in each catalytic step were first identified from the literature, and *S. sclarea* proteins containing these domains were then selected as candidates. They were further confirmed by a conserved domain analysis when aligned with validated genes from other species and the phylogenetic tree construction.

### Real-time RT-PCR

Total RNAs from the different tissues were isolated using TRIzol (Invitrogen) and treated with DNase I (Thermo Scientific) to remove contaminating DNA. Complementary DNA was synthesized from 1 µg of total RNA using the SuperScript II First-Strand Synthesis System with oligo(dT)20 primers (Invitrogen), according to the manufacturer’s instructions. Quantitative PCR was performed with MESA GREEN qPCR MasterMix Plus (Eurogentech) and 200 nM of each primer in a total volume of 10 μL. Two biological replicates and three technical replicates were analyzed using the CFX384 Real-Time PCR System (Bio-Rad) with the following cycling program: 5 min 95 °C followed by 40 cycles of 15 s at 95 °C and 1 min at 60 °C. Transcript levels were normalized to the transcript level of Ss*ACTIN3* gene. Primers used in this study are listed in Supplementary Data 3.

### Recombinant constructs for protein expression in *E. coli*

The length of chloroplast signal peptide in *SsCPS2.1* (*Ss08g007510*) and SsLPPS (*Ss08g007460*) was predicted based on DeepLoc 2.0^54^ (Supplementary Fig. 13a and Supplementary Table 12). The chloroplast signal peptide truncated *SsCPS2*.1, *SsLPPS* and the mutated gene isoforms, *SsCPS2.1m* and *SsLPPSm*, were inserted into the *E. coli* expression vector pET15b using ClonExpress II One Step Cloning kit (Vazyme, Nanjing, China). The primers used in this work are listed in Supplementary Data 3. Colonies were screened by colony PCR to check the size of the inserted gene, and those which had the correct band size were sent out for sequencing. Zxpression vectors carrying SsCPS2.1 and SsLPPS were transformed into BL21(DE3)pLYS cells *E. coli* strains for protein expression.

### Expression and purification of recombinant proteins

BL21(DE3)pLYS cells transformed with the SsCPS2.1 or SsLPPS constructs were incubated in 25 ml of Luria-Bertani medium (tryptone 10 g/L, yeast extract 5 g/L, NaCl 10 g/L) supplemented with ampicillin and chloramphenicol (50 µg/ml each) and incubated overnight at 37 °C. This pre-culture was used to inoculate 2 L of the same medium supplemented with ampicillin (50 µg/ml) and the cells were grown at 37 °C in a shaking incubator at a speed of 180 rpm until OD_600_ = 0,6. IPTG was added (0.2 mM) to induce protein expression and cells were grown for 16 h at 16 °C, harvested by centrifugation, and kept overnight at –45 °C. The frozen cells were resuspended in ice-cold 1× PBS containing 5 mM DTT and 0.5 mg/mL lysozyme at a volume of 2 mL per gram of pellet. After 30 min incubation at 4 °C, cells were disrupted by sonication and lysates clarified by centrifugation at 8000 × *g*, 4 °C for 10 min.

The supernatant was incubated overnight with Ni-NTA column (Qiagen). The resin was washed four times with 1× PBS containing 10 mM imidazole, and proteins were eluted with 1× PBS containing 200 mM imidazole and 10% glycerol. Eluted proteins were buffer-exchanged into 50 mM HEPES (pH 7.5), 150 mM NaCl, 5% glycerol, and 5 mM DTT using Amicon® Ultra centrifugal filters (30 kDa MWCO). Purified proteins were aliquoted, flash-frozen in liquid nitrogen, and stored at −80 °C.

### In vitro enzyme assays and GC-MC analysis

Enzyme assays were conducted in a final volume of 500 µL assay buffer (50 mM HEPES, pH 7.5; 150 mM NaCl; 7.5 mM MgCl2; 20 µM MnCl2; 5% glycerol; 5 mM DTT), containing 100 µg purified protein and 20 µM geranylgeranyl pyrophosphate (GGPP; Sigma-Aldrich). Reactions were incubated with gentle shaking for 1 h at 30 °C in the dark. Reaction products were dephosphorylated by the addition of 7 U alkaline phosphatase (Invitrogen) and incubation overnight at 37 °C. Reactions were extracted with 500 µL hexanes containing 40 µM 10-undecen-1-ol (internal standard). After centrifugation at 1000 × *g*, 4 °C for 20 min, the upper organic layer was subjected to GC-MS analysis on a 7890B/5977 A GC-MS system from Agilent. The system was equipped with a 10 m guard column and an Rxi-5Sil MS column (length, 30 m; inner diameter, 0.25 mm; film thickness, 0.25 μm) (Restek, Bellefonte, PA, USA). One µl of sample was injected with a split ratio of 30:1. Oven temperature was set to 110 °C for 2 min, then increased to 270 °C at a rate of 10 °C/min, and finally set to 270 °C for 2 min. Other temperatures were set as follows: injector, 270 °C; transfer line, 270 °C; source, 230 °C; quadrupole, 150 °C. The carrier gas was helium at a constant flow of 1 mL/min. The quadrupole mass spectrometer was switched on after a solvent delay of 5 min and was programmed to scan from *m/z* 35 to *m/z* 350.

The GC-MS chromatograms were carried out with the MassHunter Quantitative Analysis software from Agilent. Peaks identification was conducted using commercial MS libraries, including the Flavour & Fragrance Natural & Synthetic Compounds GCMS library (FFNSC 2)^55^ and the National Institute of Standards & Technology, Wiley (NIST) mass spectral library. Retention index (RI) values were calculated and compared against their reported values in the GC-MS mass spectral libraries, FFNSC 2 and NIST, and in the scientific literature^56–59^ (Supplementary Table 13).

### Reporting summary

Further information on research design is available in the Nature Portfolio Reporting Summary linked to this article.

## Supplementary information

Supplementary Information

Peer Review file

Description of Additional Supplementary Files

Supplementary Data 1

Supplementary Data 2

Supplementary Data 3

Reporting Summary

## Source data

Source Data

## Data Availability

The raw sequencing data generated in this study have been deposited in the NCBI Sequence Read Archive (SRA) under BioProject accession PRJNA1417963 and PRJNA1337316 [https://www.ncbi.nlm.nih.gov/bioproject/PRJNA1337316]. The *S. sclarea* genome assembly (FASTA) and the corresponding gene annotation files (GFF3) are publicly available at French Government Data Research Repository [10.57745/RE3LLC]. Source data are provided with this paper.

## References

[1] Wink, M. Evolution of secondary metabolites from an ecological and molecular phylogenetic perspective. *Phytochemistry* **64**, 3–19 (2003). 12946402 10.1016/s0031-9422(03)00300-5

[2] Mint Evolutionary Genomics Consortium. Phylogenomic mining of the mints reveals multiple mechanisms contributing to the evolution of chemical diversity in Lamiaceae. *Mol. Plant* **11**, 1084–1096 (2018). 10.1016/j.molp.2018.06.002 29920355

[3] Dudareva, N., Klempien, A., Muhlemann, J. K. & Kaplan, I. Biosynthesis, function and metabolic engineering of plant volatile organic compounds. *N. Phytol.* **198**, 16–32 (2013). 10.1111/nph.12145 23383981

[4] Moshari-Nasirkandi, A., Alirezalu, A., Alipour, H. & Amato, J. Screening of 20 species from Lamiaceae family based on phytochemical analysis, antioxidant activity and HPLC profiling. *Sci. Rep.* **13**, 16987 (2023). 37813985 10.1038/s41598-023-44337-7 PMC10562417

[5] Chalvin, C. et al. Sclareol and linalyl acetate are produced by glandular trichomes through the MEP pathway. *Hortic. Res.* **8**, 206 (2021). 34593779 10.1038/s41438-021-00640-w PMC8484277

[6] Caniard, A. et al. Discovery and functional characterization of two diterpene synthases for sclareol biosynthesis in *Salvia sclarea* and their relevance for perfume manufacture. *BMC Plant Biol.* **12**, 119 (2012). 22834731 10.1186/1471-2229-12-119 PMC3520730

[7] Choi, S., Kang, Y. & Kim, C. Chromosome-level genome assembly of *Salvia sclarea*. *Sci. Data* **12**, 14 (2025). 39755684 10.1038/s41597-024-04347-0 PMC11700129

[8] Wlodzimierz, P., Hong, M. & Henderson, I. R. TRASH: tandem repeat annotation and structural hierarchy. *Bioinformatics* **39**, btad308 (2023). 37162382 10.1093/bioinformatics/btad308 PMC10199239

[9] Muravenko, O. V. et al. Integration of Repeatomic and cytogenetic data on satellite DNA for genome analysis in *Salvia*. *Plants* **11**, 2244 (2022). 36079625 10.3390/plants11172244 PMC9460151

[10] Ou, S., Chen, J. & Jiang, N. Assessing genome assembly quality using the LTR Assembly Index (LAI). *Nucleic Acids Res.* **46**, e126 (2018). 30107434 10.1093/nar/gky730 PMC6265445

[11] Mizutani, M. & Ohta, D. Diversification of P450 genes during land plant evolution. *Annu. Rev. Plant Biol.* **61**, 291–315 (2010). 20192745 10.1146/annurev-arplant-042809-112305

[12] Hansen, C. C., Nelson, D. R., Møller, B. L. & Werck-Reichhart, D. Plant cytochrome P450 plasticity and evolution. *Mol. Plant* **14**, 1244–1265 (2021). 34216829 10.1016/j.molp.2021.06.028

[13] Zheng, X., Li, P. & Lu, X. Research advances in cytochrome P450-catalysed pharmaceutical terpenoid biosynthesis in plants. *J. Exp. Bot.* **70**, 4619–4630 (2019). 31037306 10.1093/jxb/erz203

[14] Xiong, X. et al. The Taxus genome provides insights into paclitaxel biosynthesis. *Nat. Plants* **7**, 1026–1036 (2021). 34267359 10.1038/s41477-021-00963-5 PMC8367818

[15] Jia, Q. et al. Origin and early evolution of the plant terpene synthase family. *Proc. Natl. Acad. Sci. USA* **119**, e2100361119 (2022). 35394876 10.1073/pnas.2100361119 PMC9169658

[16] Köksal, M., Potter, K., Peters, R. J. & Christianson, D. W. 1.55Å-resolution structure of ent-copalyl diphosphate synthase and exploration of general acid function by site-directed mutagenesis. *Biochim. Biophys. Acta* **1840**, 184–190 (2014). 24036329 10.1016/j.bbagen.2013.09.004 PMC3859867

[17] Jumper, J. et al. Highly accurate protein structure prediction with AlphaFold. *Nature* **596**, 583–589 (2021). 34265844 10.1038/s41586-021-03819-2 PMC8371605

[18] Günnewich, N. et al. A diterpene synthase from clary sage catalyzes cyclization of geranylgeranyl diphosphate to (8R)-hydroxy-copalyl diphosphate. *Phytochemistry* **91**, 93–99 (2013). 22959531 10.1016/j.phytochem.2012.07.019

[19] Okada, K., Abe, H. & Arimura, G. Jasmonates induce both defense responses and communication in monocots and dicots. *Plant Cell Physiol.* **56**, 16–27 (2015). 25378688 10.1093/pcp/pcu158

[20] Santner, A. & Estelle, M. The JAZ proteins link jasmonate perception with transcriptional changes. *Plant Cell* **19**, 3839–3842 (2007). 18165326 10.1105/tpc.107.056960 PMC2217635

[21] Wong, D. C. J., Pichersky, E. & Peakall, R. Specialized metabolite diversity in plant-pollinator interactions. *Curr. Opin. Plant Biol.* **73**, 102332 (2023). 36652780 10.1016/j.pbi.2022.102332

[22] de Castro, ÉC. P., Musgrove, J., Bak, S., McMillan, W. O. & Jiggins, C. D. Phenotypic plasticity in butterfly chemical defense allows diverse host usage. *Biol. Lett.* **17**, 20200863 (2021). 33784874 10.1098/rsbl.2020.0863 PMC8086984

[23] Nützmann, H. W., Huang, A. & Osbourn, A. Plant metabolic clusters—from genetics to genomics. *N. Phytol.* **211**, 771–789 (2016). 10.1111/nph.13981 PMC5449196 27112429

[24] Kerwin, R. E. et al. Tomato root specialized metabolites evolved through gene duplication and regulatory divergence. *Sci. Adv.* **10**, eadn3991 (2024). 38657073 10.1126/sciadv.adn3991 PMC11094762

[25] Li, D., Halitschke, R., Baldwin, I. T. & Gaquerel, E. Information theory tests critical predictions of plant defense theory for specialized metabolism. *Sci. Adv.* **6**, eaaz0381 (2020). 32577508 10.1126/sciadv.aaz0381 PMC7286674

[26] Del Carratore, F. et al. Computational identification of co-evolving multi-gene modules in microbial BGCs. *Commun. Biol.* **2**, 83 (2019). 30854475 10.1038/s42003-019-0333-6 PMC6395733

[27] Jensen, P. R. Natural products and the gene cluster revolution. *Trends Microbiol.* **24**, 968–977 (2016). 27491886 10.1016/j.tim.2016.07.006 PMC5123934

[28] Pichot, C. et al. Cantaloupe melon genome reveals 3D chromatin features and structural relationships. *iScience* **25**, 103696 (2021). 35059606 10.1016/j.isci.2021.103696 PMC8760558

[29] Liu, C. In situ Hi-C library preparation for plants. *Methods Mol. Biol.* **1629**, 155–166 (2017). 28623585 10.1007/978-1-4939-7125-1_11

[30] Buenrostro, J. D., Wu, B., Chang, H. Y. & Greenleaf, W. J. ATAC-seq: a method for assaying chromatin accessibility genome-wide. *Curr. Protoc. Mol. Biol.* **109**, 21–29 (2015). 10.1002/0471142727.mb2129s109 PMC4374986 25559105

[31] Servant, N. et al. HiC-Pro: an optimized and flexible pipeline for Hi-C data processing. *Genome Biol.* **16**, 259 (2015). 26619908 10.1186/s13059-015-0831-x PMC4665391

[32] Zhou, C., McCarthy, S. A. & Durbin, R. YaHS: yet another Hi-C scaffolding tool. *Bioinformatics* **39**, btac808 (2023). 36525368 10.1093/bioinformatics/btac808 PMC9848053

[33] Brown, M. R., Gonzalez de La Rosa, P. M. & Blaxter, M. tidk: a toolkit to rapidly identify telomeric repeats. *Bioinformatics* **41**, btaf049 (2025). 39891350 10.1093/bioinformatics/btaf049 PMC11814493

[34] Manni, M., Berkeley, M. R., Seppey, M., Simão, F. A. & Zdobnov, E. M. BUSCO update: novel and streamlined workflows with broader phylogenetic coverage. *Mol. Biol. Evol.* **38**, 4647–4654 (2021). 34320186 10.1093/molbev/msab199 PMC8476166

[35] Ou, S. et al. Benchmarking transposable element annotation methods. *Genome Biol.* **20**, 275 (2019). 31843001 10.1186/s13059-019-1905-y PMC6913007

[36] Ou, S. & Jiang, N. L. T. R. _retriever: identification of long terminal repeat retrotransposons. *Plant Physiol* **176**, 1410–1422 (2018). 29233850 10.1104/pp.17.01310 PMC5813529

[37] Foissac, S. et al. Genome annotation in plants and fungi: EuGene as a model platform. *Curr. Bioinform.* **3**, 87–97 (2008).

[38] Stoesser, G., Sterk, P., Tuli, M. A., Stoehr, P. J. & Cameron, G. N. The EMBL nucleotide sequence database. *Nucleic Acids Res.* **25**, 7–13 (1997). 9016493 10.1093/nar/25.1.7 PMC146376

[39] Lamesch, P. et al. The Arabidopsis Information Resource (TAIR): improved annotation and new tools. *Nucleic Acids Res.* **40**, 1202–1210 (2012). 10.1093/nar/gkr1090 PMC3245047 22140109

[40] Jones, P. et al. InterProScan 5: genome-scale protein function classification. *Bioinformatics* **30**, 1236–1240 (2014). 24451626 10.1093/bioinformatics/btu031 PMC3998142

[41] Huerta-Cepas, J. et al. eggNOG 5.0: orthology resource with functional and phylogenetic annotation. *Nucleic Acids Res.* **47**, D309–D314 (2019). 30418610 10.1093/nar/gky1085 PMC6324079

[42] Dai, X., Sinharoy, S., Udvardi, M. & Zhao, P. X. PlantTFcat: an online plant transcription factor categorization tool. *BMC Bioinform.* **14**, 321 (2013). 10.1186/1471-2105-14-321 PMC4225725 24219505

[43] Benson, G. Tandem repeats finder: a program to analyze DNA sequences. *Nucleic Acids Res.* **27**, 573–580 (1999). 9862982 10.1093/nar/27.2.573 PMC148217

[44] Keilwagen, J. & Grau, J. TRASH: tandem repeat annotation and search. *Bioinformatics* **36**, 2255–2257 (2020).

[45] Vollger, M. R., Kerpedjiev, P., Phillippy, A. M. & Eichler, E. E. StainedGlass: visualization of tandem repeat structures. *Bioinformatics* **38**, 2049–2051 (2022). 35020798 10.1093/bioinformatics/btac018 PMC8963321

[46] Emms, D. M. & Kelly, S. OrthoFinder: phylogenetic orthology inference. *Genome Biol.* **20**, 238 (2019). 31727128 10.1186/s13059-019-1832-y PMC6857279

[47] Yang, Z. PAML 4: phylogenetic analysis by maximum likelihood. *Mol. Biol. Evol.* **24**, 1586–1591 (2007). 17483113 10.1093/molbev/msm088

[48] Xu, S. et al. Using clusterProfiler to characterize multiomics data. *Nat. Protoc.* **19**, 3292–3320 (2024). 39019974 10.1038/s41596-024-01020-z

[49] Wang, Y. et al. MCScanX: toolkit for analysis of gene synteny and collinearity. *Nucleic Acids Res.* **40**, e49 (2012). 22217600 10.1093/nar/gkr1293 PMC3326336

[50] Tang, H. et al. JCVI: a versatile toolkit for comparative genomics analysis. *iMeta* **3**, e211 (2024). 10.1002/imt2.211 PMC11316928 39135687

[51] Nelson, D. R. The cytochrome P450 homepage. *Hum. Genomics* **4**, 59–65 (2009). 19951895 10.1186/1479-7364-4-1-59 PMC3500189

[52] Durst, F. & Nelson, D. R. Diversity and evolution of plant P450s and P450-reductases. *Drug Metab. Drug Interact.* **12**, 189–206 (1995). 10.1515/dmdi.1995.12.3-4.189 8820852

[53] Kumar, S., Stecher, G., Li, M., Knyaz, C. & Tamura, K. MEGA X: molecular evolutionary genetics analysis across computing platforms. *Mol. Biol. Evol.* **35**, 1574–1549 (2018). 10.1093/molbev/msy096 PMC5967553 29722887

[54] Thumuluri, V., Almagro Armenteros, J. J., Johansen, A. R., Nielsen, H. & Winther, O. DeepLoc 2.0: multi-label subcellular localization prediction using protein language models. *Nucleic Acids Res.* **50**, W228–W234 (2022). 35489069 10.1093/nar/gkac278 PMC9252801

[55] Mondello, L. *Flavors and Fragrances of Natural and Synthetic Compounds, Mass Spectral Database* (FFNSC 2, John Wiley & Sons Inc., New York, NY, USA, 2011).

[56] Adams, R. P. *Identification of Essential Oil Components by Gas Chromatography/Mass Spectroscopy.* 4th ed. (Allured Pub. Corp., 2007).

[57] Papanikolaou, A. S. et al. Chemical and transcriptomic analyses of leaf trichomes from Cistus creticus subsp. creticus reveal the biosynthetic pathways of certain labdane-type diterpenoids and their acetylated forms. *J. Exp. Bot.* **75**, 3431–3451 (2024). 38520311 10.1093/jxb/erae098 PMC11156806

[58] Kuźma, L. et al. Chemical composition and biological activities of essential oil from *Salvia sclarea* plants regenerated in vitro. *Molecules* **14**, 1438–1447 (2009). 19384275 10.3390/molecules14041438 PMC6254371

[59] McCadden, C. A. et al. Discovery of a plant-like tridomain bifunctional syn-abieta-7,13-diene synthase in Streptomyces. *Org. Biomol. Chem.* **23**, 9845–9850 (2025). 40511979 10.1039/d5ob00724k PMC12164552

