# Supplementary material for: Neofunctionalization underlies the evolutionary origin of sclareol biosynthesis in the mint family
Source: Nat Commun. 2026 May 22;17:6745. doi: 10.1038/s41467-026-73637-5 (PMC13385783; doi:10.1038/s41467-026-73637-5)
Supplement: Supplementary file 1 — Supplementary Information [file 41467_2026_73637_MOESM1_ESM.pdf]

Neofunctionalization underlies the evolutionary origin of sclareol biosynthesis in the mint family.

Dong *et al.*

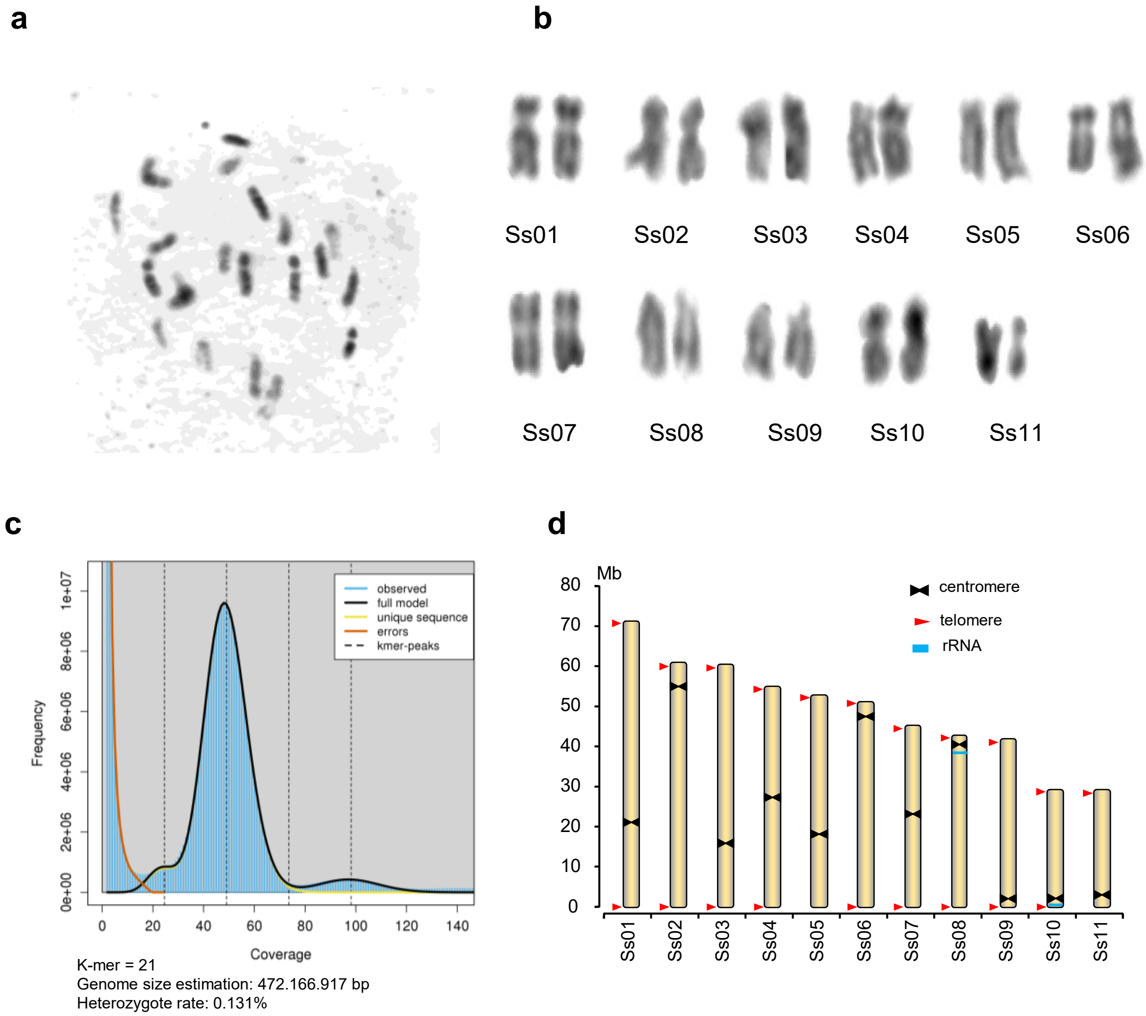

Supplementary Fig. 1. Chromosome counting and genome size estimation of *S. sclarea*. (**a**) Mitotic metaphase chromosomes from *S. sclarea* root tip cells stained with orcein. (**b**) Karyotype of *S. sclarea* showing 11 pairs of bivalent chromosomes (Ss01–Ss11). (**c**) Genome size estimation based on 21-Kmer frequency distribution derived from a genomic survey. The major peak represents the haploid genome coverage, with an estimated genome size of ~472 Mb and a heterozygosity rate of 0.131%. (**d**) Chromosomal features of the *S. sclarea* genome. The eleven chromosomes (Ss01–Ss11) are represented as vertical bars scaled by size (in Mb). Black triangles mark centromere positions, red arrowheads indicate telomeric repeats, and cyan bars denote rRNA gene clusters. Chromosomes are arranged and numbered in order of decreasing size.

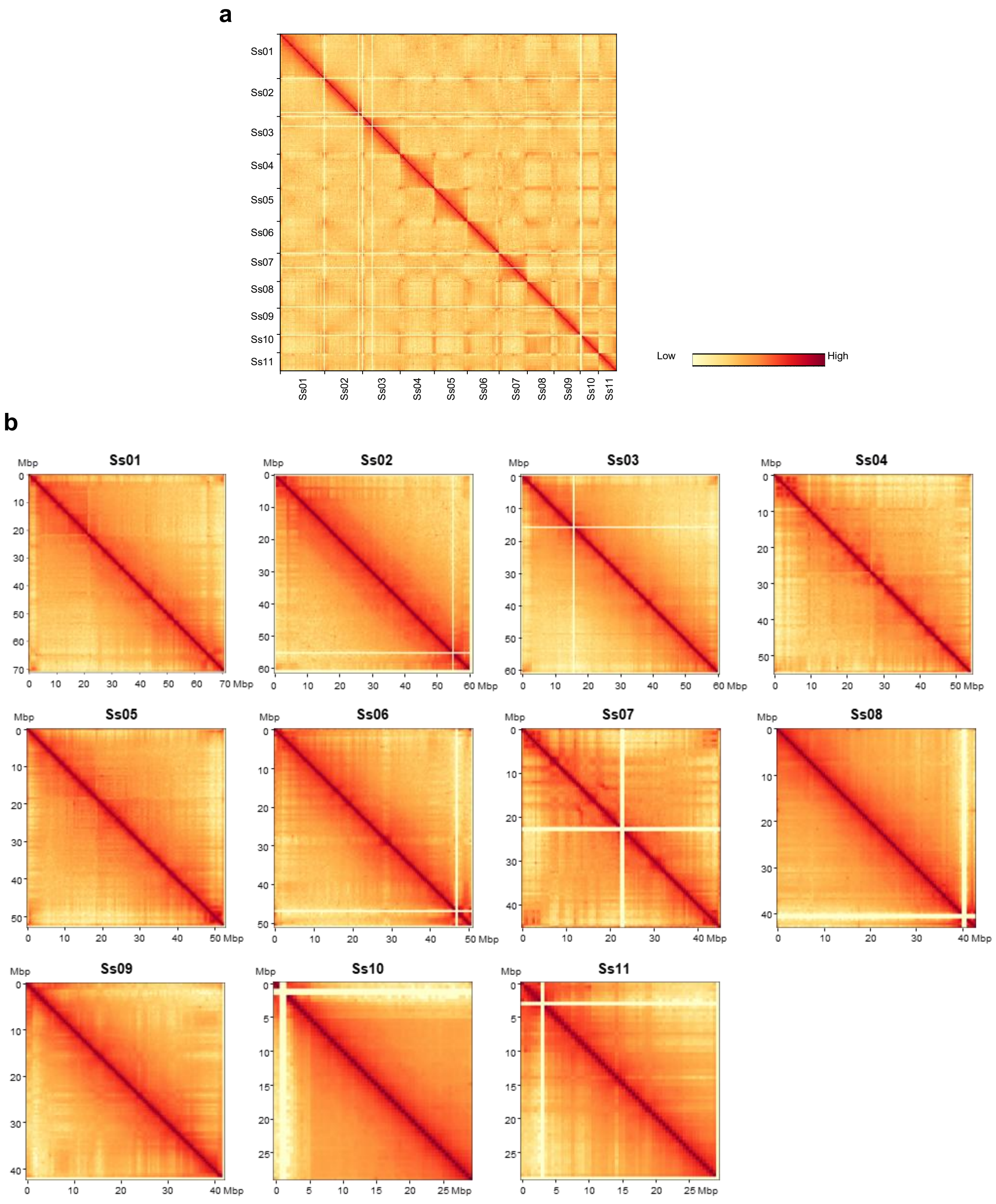

Supplementary Fig. 2. Chromatin interaction landscape of the *S. sclarea* genome assembly at 500-kbp resolution. (**a**) Genome-wide Hi-C contact matrix showing interaction frequencies between all 11 chromosomes. (**b**) Intra-chromosomal Hi-C contact maps for individual chromosomes (Ss01–Ss11). Heatmap intensity reflects the frequency of Hi-C contacts between 500-kbp genomic bins, plotted on a logarithmic scale. The color bar to the right indicates interaction intensity levels, with white representing low and dark red representing high contact probabilities.

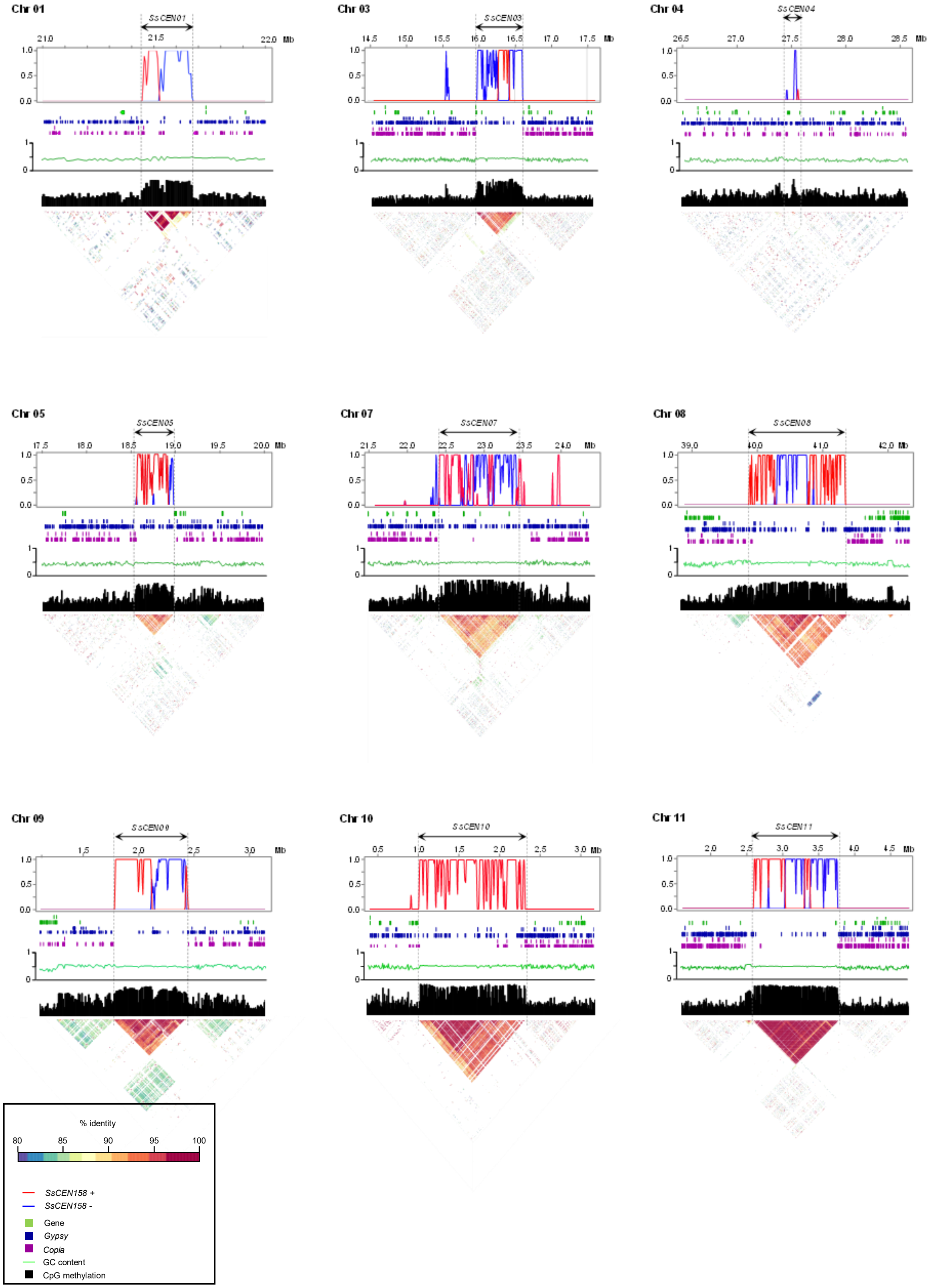
Chr 01
SsCEN01
Chr 03
SsCEN03
Chr 04
SsCEN04
Chr 05
SsCEN05
Chr 07
SsCEN07
Chr 08
SsCEN08
Chr 09
SsCEN09
Chr 10
SsCEN10
Chr 11
SsCEN11
Mb
% identity
80
85
90
95
100
SsCEN158 +
SsCEN158 -
Gene
Gypsy
Copia
GC content
CpG methylation

Supplementary Fig. 3. Structural and epigenetic features of the centromeric regions in the *S. sclarea* genome. The distributions of *CEN158* per 10-kbp on forward (red) or reverse (blue) strands, genes (green), *Gypsy* LTR (dark blue), *Copia* LTR (purple), GC content (light green line), CpG methylation pattern (black bar plot) and *CEN158* sequence similarity on the centromeric regions were plotted successively.

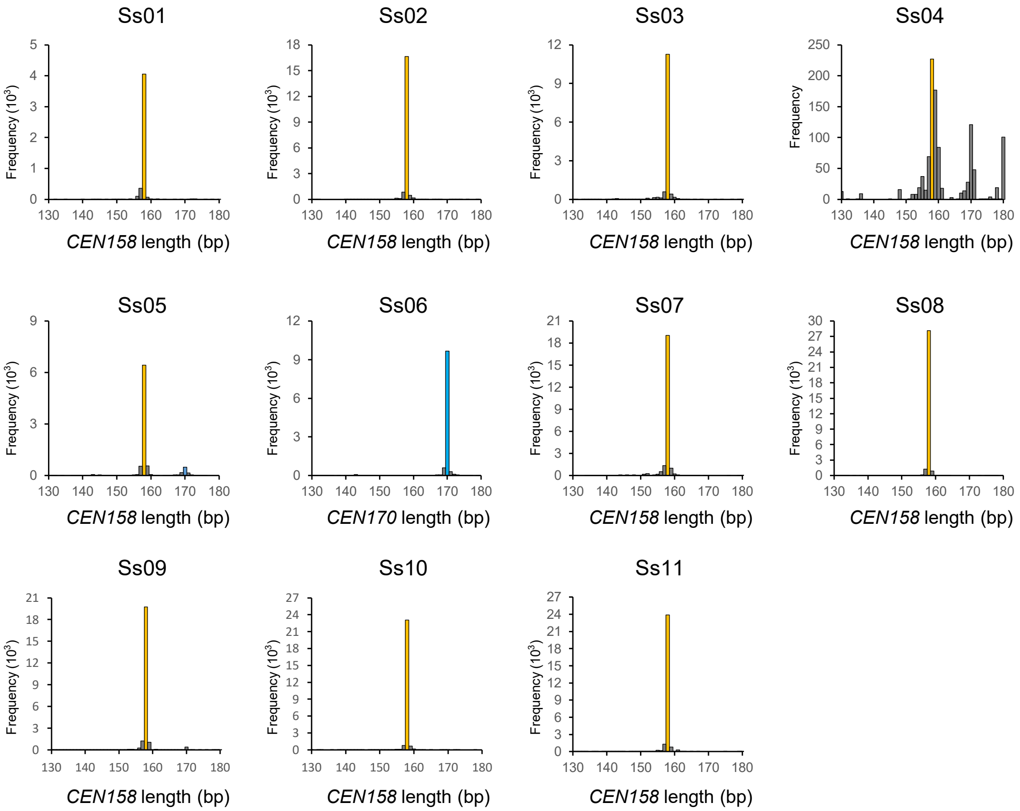

Supplementary Fig. 4. Monomer length distribution of *CEN158* satellite repeats across *S. sclarea* chromosomes. Histograms show the length (in base pairs) of *CEN158* satellite repeat monomers for each chromosome (Ss01-Ss11). Most chromosomes display a highly conserved monomer length centered around ~158 bp. Notably, chromosome Ss06 includes *CEN170* repeats, with a distinct length distribution.

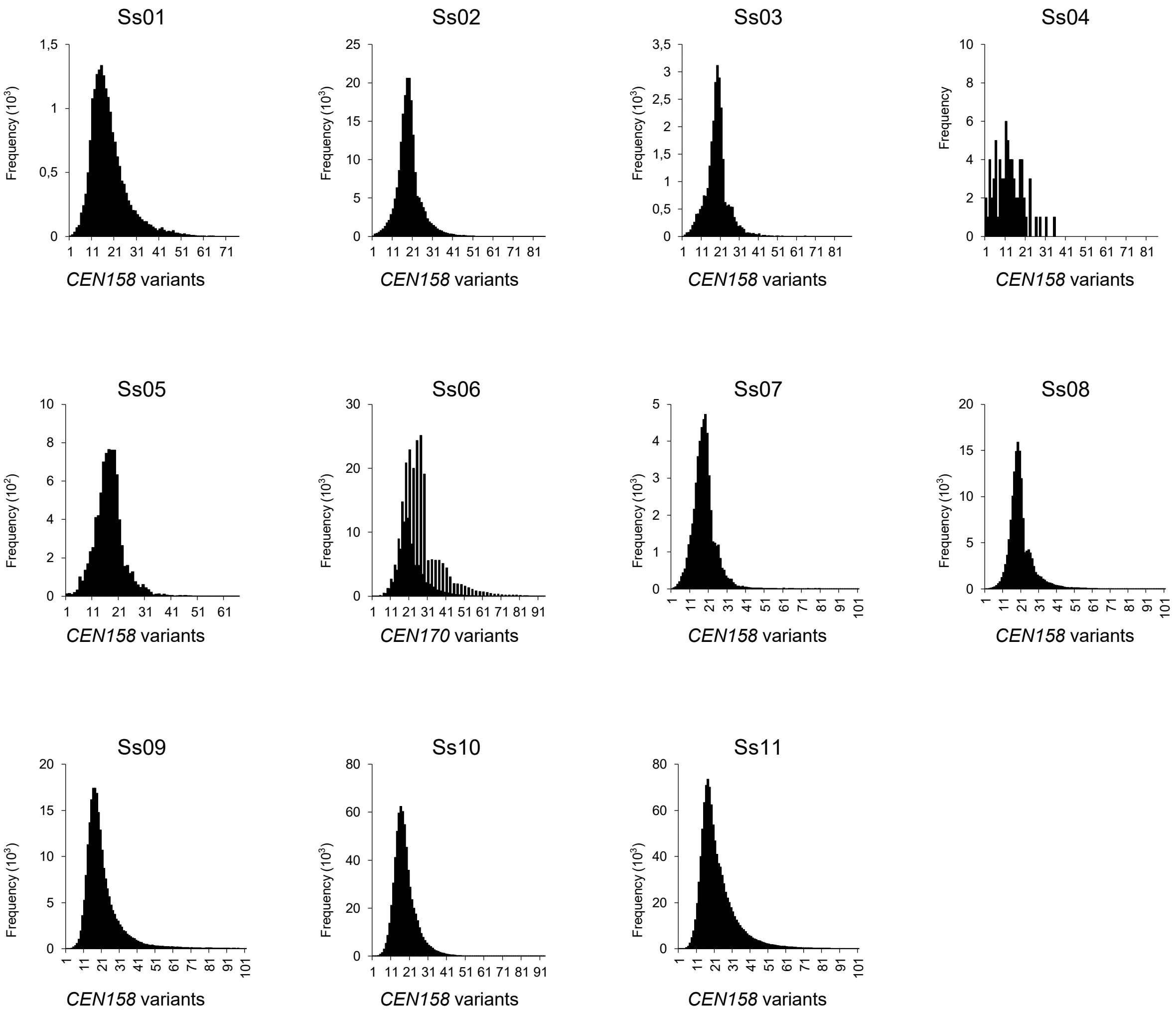

Supplementary Fig. 5. Distribution of *CEN158* sequence variants across the chromosomes of *S. sclarea*. Histograms represent the frequency of *CEN158* variants identified on each chromosome (Ss01-Ss11), relative to the genome-wide *CEN158* consensus sequence. Variation patterns reflect the sequence diversity of centromeric repeats within and across chromosomes. *CEN170* variants are observed on chromosome Ss06.

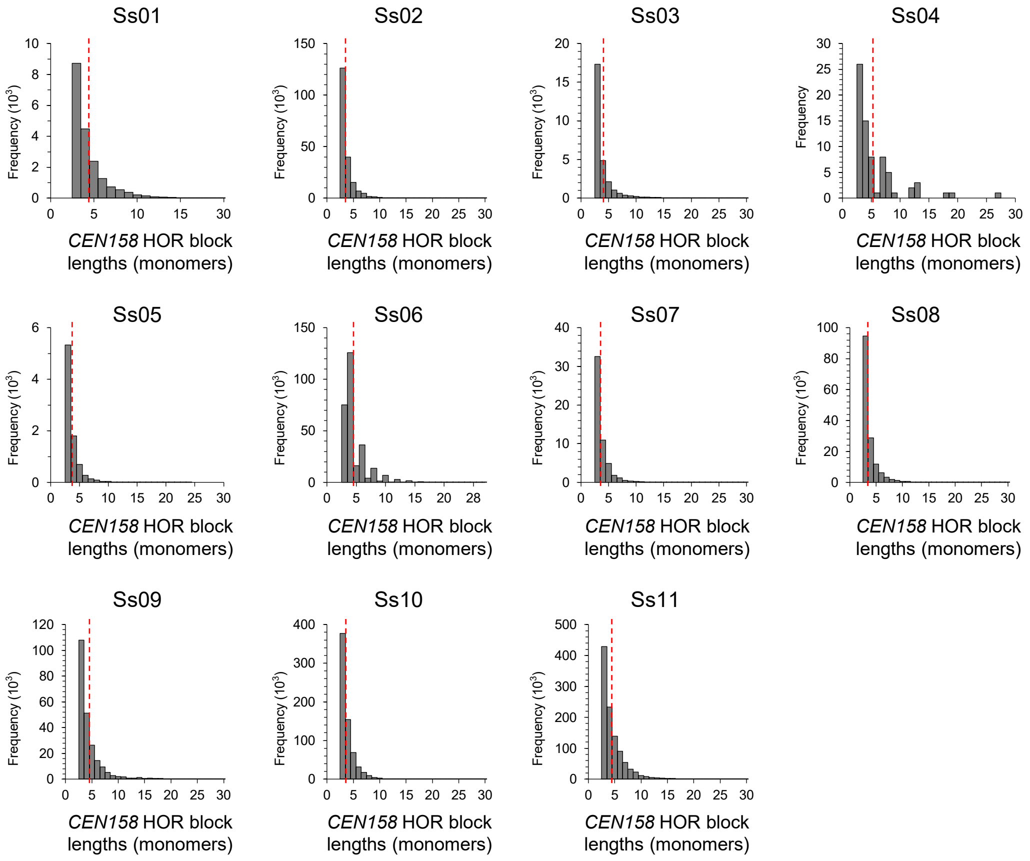

Supplementary Fig. 6. Distribution of CEN158 higher-order repeat (HOR) block lengths in the centromeres of the *S. sclarea* genome. Histograms show the frequency of *CEN158* HOR block lengths (in number of monomers) for each chromosome (Ss01-Ss11). The red dotted line indicates the mean HOR block length per chromosome.

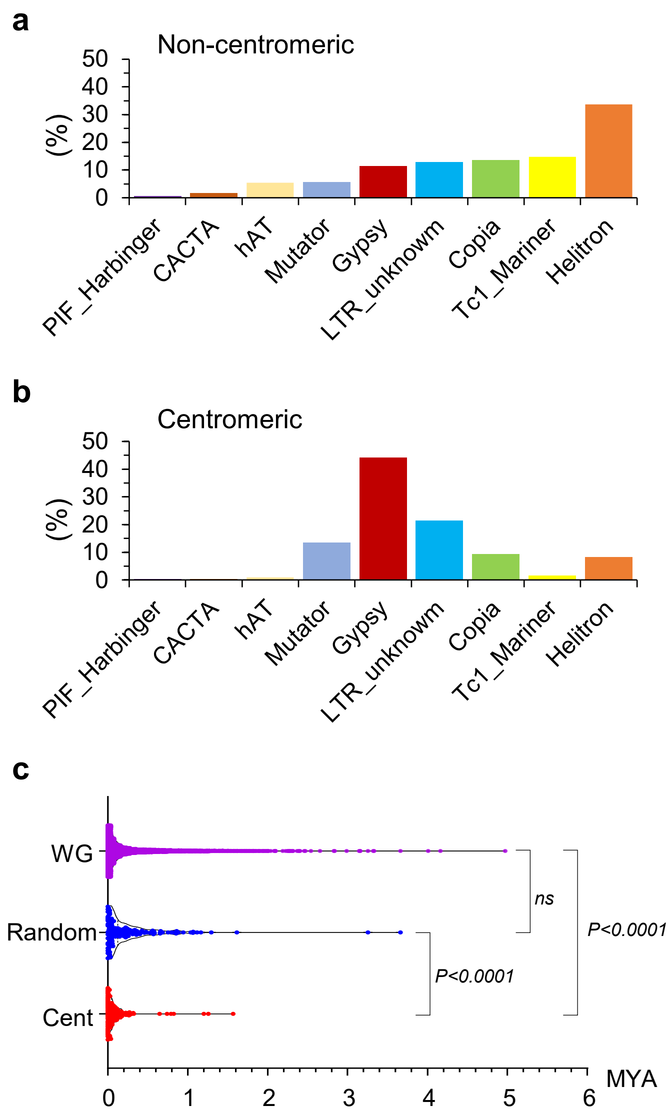

Supplementary Fig. 7. Characterization of repetitive elements in centromeric regions of *S. sclarea*. (**a, b**) Proportional content of transposable elements in non-centromeric (**a**) and centromeric (**b**) regions of *S. sclarea* genome. Helitrons dominate the non-centromeric regions, while Gypsy LTR retrotransposons are the most abundant in centromeric regions. (**c**) Insertion time estimates (in million years ago, MYA) for LTR retrotransposons located in whole-genome (WG), random, and centromeric (Cent) regions. LTRs in centromeric regions show significantly more recent insertion times compared to random and whole-genome distributions. Statistical comparisons were made using two-tailed Student's t-test, with p-values indicated.

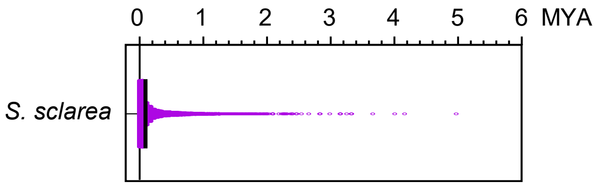

Supplementary Fig. 8. Insertion time distribution of intact LTR retrotransposons in the *S. sclarea* genome. Violin plot showing the estimated insertion times (in million years ago, MYA) of intact LTR retrotransposons across the whole genome of *S. sclarea*.

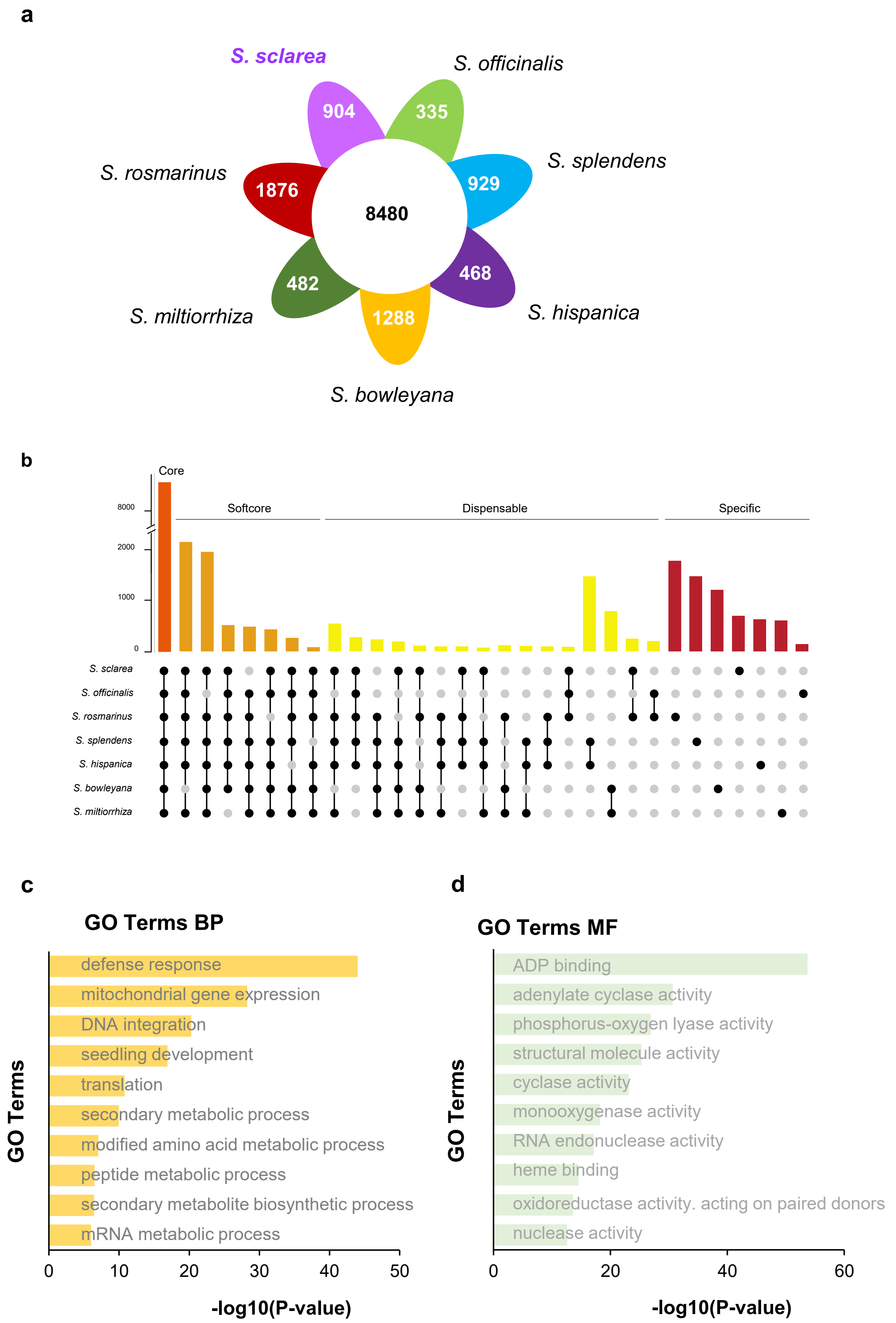
a
S. sclarea
S. officinalis
S. splendens
S. hispanica
S. bowleyana
S. miltiorrhiza
S. rosmarinus
904
335
929
468
1288
482
1876
8480
b
Core
Softcore
Dispensable
Specific
8000
2000
1000
0
S. sclarea
S. officinalis
S. rosmarinus
S. splendens
S. hispanica
S. bowleyana
S. miltiorrhiza
c
GO Terms BP
GO Terms
defense response
mitochondrial gene expression
DNA integration
seedling development
translation
secondary metabolic process
modified amino acid metabolic process
peptide metabolic process
secondary metabolite biosynthetic process
mRNA metabolic process
0 10 20 30 40 50
-log10(P-value)
d
GO Terms MF
GO Terms
ADP binding
adenylate cyclase activity
phosphorus-oxygen lyase activity
structural molecule activity
cyclase activity
monooxygenase activity
RNA endonuclease activity
heme binding
oxidoreductase activity. acting on paired donors
nuclease activity
0 20 40 60
-log10(P-value)

Supplementary Fig. 9. Comparative genome analysis of *Salvia* species. (**a**) Flower plot showing the number of shared and unique gene families among seven *Salvia* species. The central number (8,480) represents core gene families shared by all species. Petals indicate species-specific gene families, with *S. sclarea* contributing 904 unique families. (**b**) UpSet plot showing the distribution of core (present in all 7 genomes), softcore (6 genomes), dispensable (2 to 5 genomes), and species-specific (1 genome) gene families. (**c**) Gene Ontology (GO) enrichment analysis of significantly expanded gene families in *S. sclarea*, biological process (BP) terms. (**d**) GO enrichment for molecular function (MF) terms. Enrichment was assessed using one-sided Fisher's exact tests, and *p*-values were corrected for multiple comparisons using the Benjamini–Hochberg method.

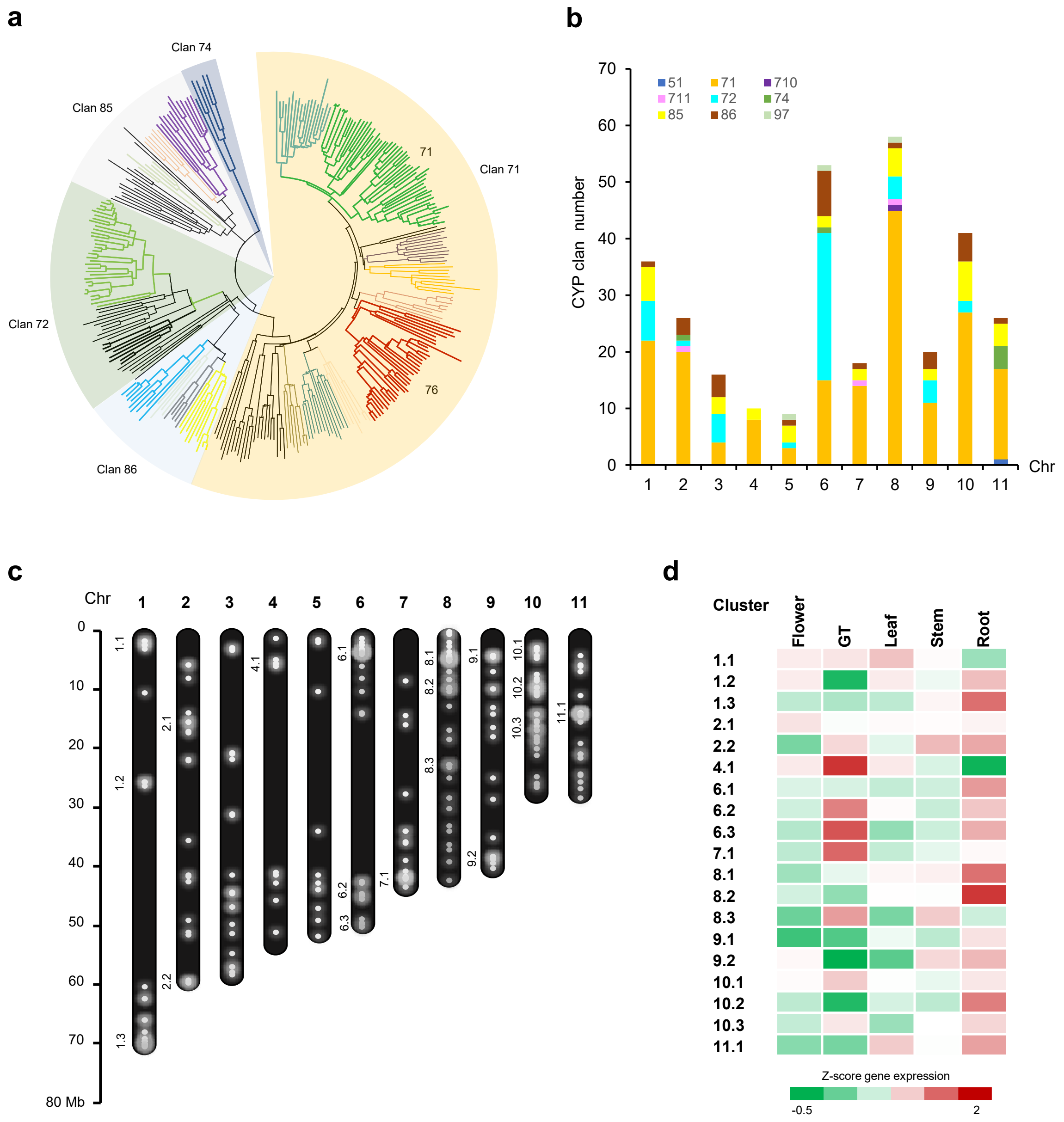

Supplementary Fig. 10. Genomic distribution, phylogeny, and tissue-specific expression of *S. sclarea* cytochrome P450 (CYP450) genes. (**a**) Maximum-likelihood phylogenetic tree of *S. sclarea* CYP450 proteins, grouped into clans (e.g., clans 71, 72, 85, etc.). (**b**) Number and clan distribution of CYP450 genes across the 11 chromosomes. CYP71 and CYP72 clans (in orange and blue, respectively) are predominantly located on chromosomes 8 and 6. (**c**) Genomic positions of CYP450 genes mapped onto the *S. sclarea* chromosomes. Gene clusters (defined as ≥4 CYP450s and ≤1.5 Mb of gene spacing between two adjacent CYP450s) are labeled at their chromosomal locations. (**d**) Heatmap showing tissue-specific expression profiles of CYP450 gene clusters in flower, glandular trichomes (GT), leaf, stem, and root. Cluster expression represents the summed expression of all CYP450s within each cluster.

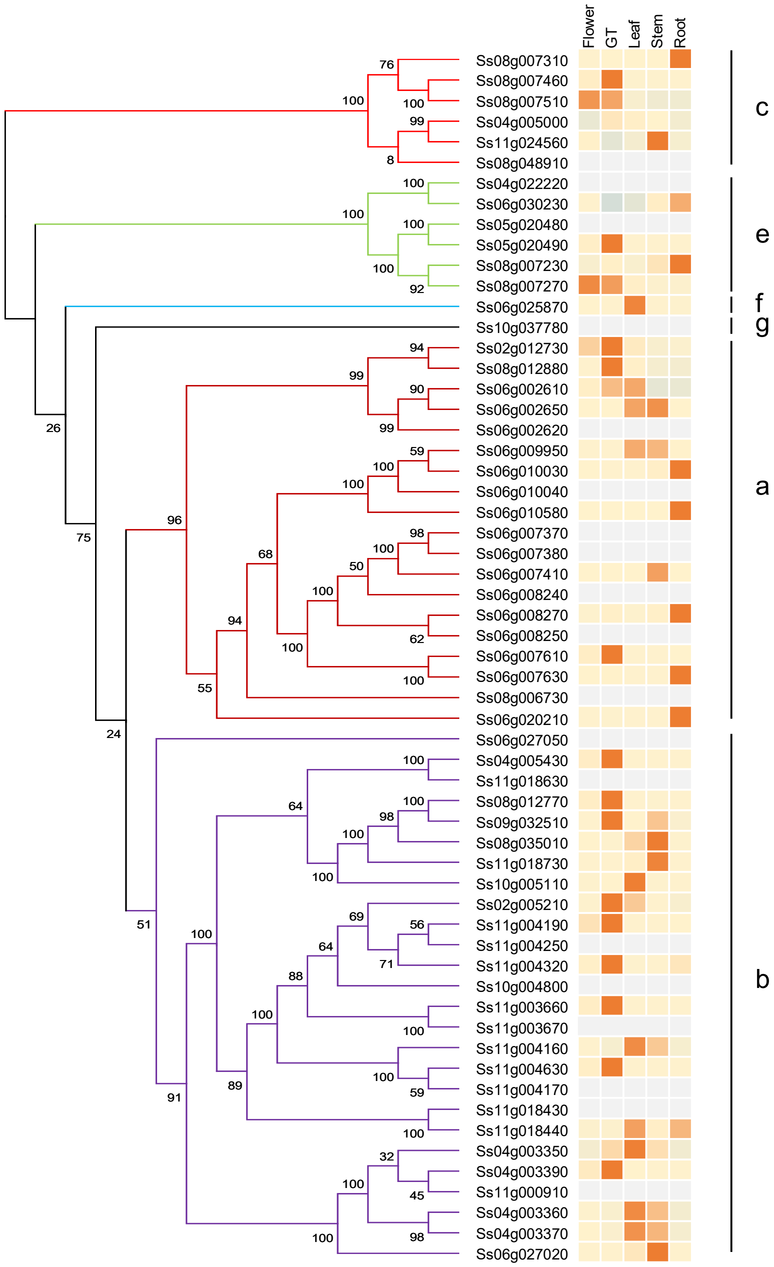

Supplementary Fig. 11. Phylogeny and tissue-specific expression of *S. sclarea* terpene synthase (TPS) genes. Maximum-likelihood phylogenetic tree of *S. sclarea* TPS proteins, classified into subfamilies (a, b, c, e, f, g, and h). Bootstrap support values are indicated at the nodes. The accompanying heatmap displays the tissue-specific expression profiles of TPS genes across flower, glandular trichomes (GT), leaf, stem, and root.

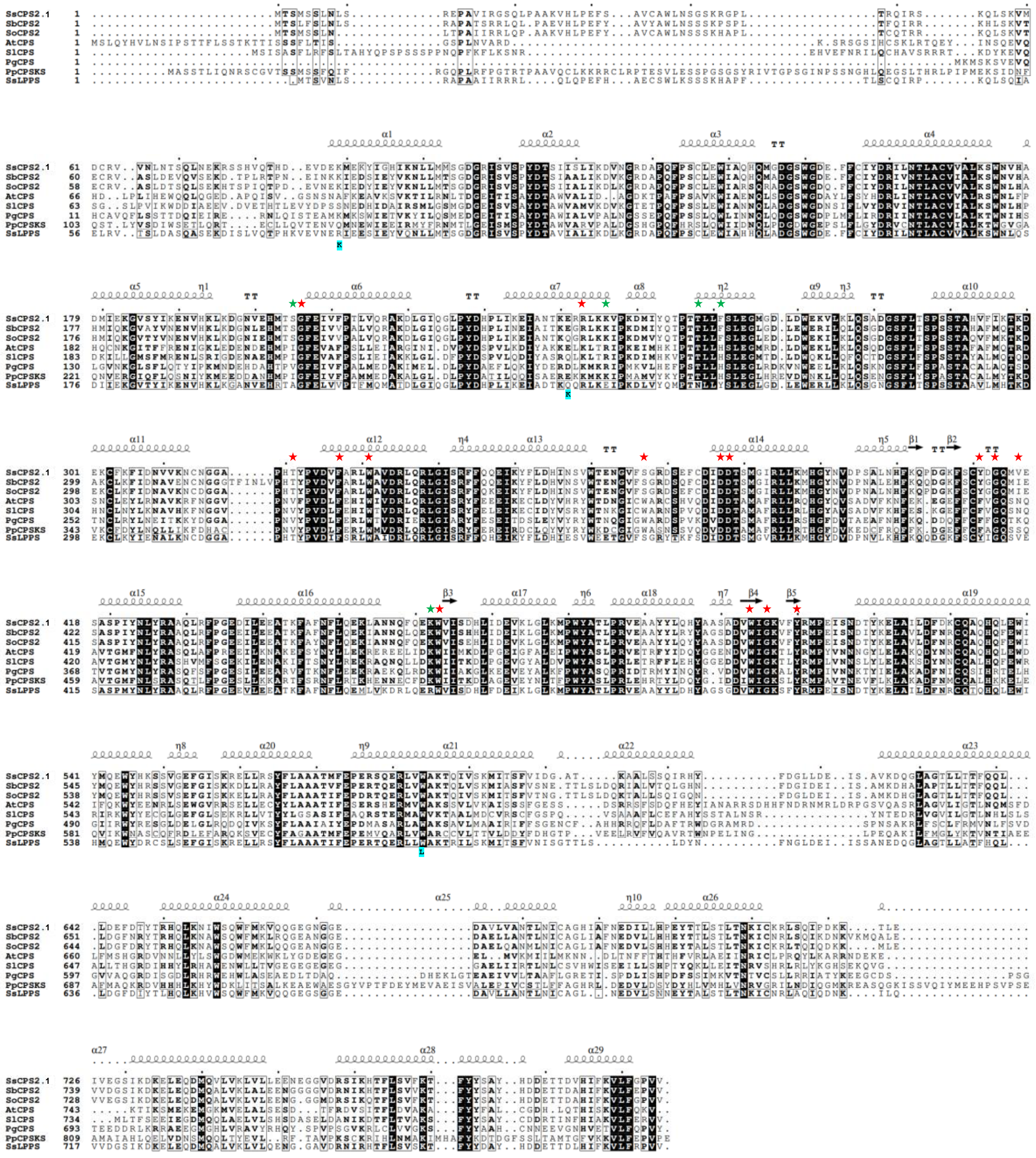

Supplementary Fig. 12. Multiple sequence alignment of class II diTPS proteins with ent-copalyl diphosphate synthase (eCPS) activity. Amino acid alignment of *S. sclarea* SsLPPS and SsCPS2.1 isoforms with homologous diTPS proteins from *S. bowleyana* (Sb), *S. officinalis* (So), *Arabidopsis thaliana* (At), *Solanum lycopersicum* (Sl), *Picea glauca* (Pg), and *Physcomitrella patens* (Pp). Residue numbers the alignment correspond to positions in the aligned sequences. Conserved amino acids are highlighted in black. Red stars indicate active-site residues conserved between SsLPPS and SsCPS2.1; green stars denote polymorphic residues within the active site between the two *S. sclarea* isoforms. Residues highlited in blue correspond to polymorphic residues identified in SsLPPS previously reported[6].

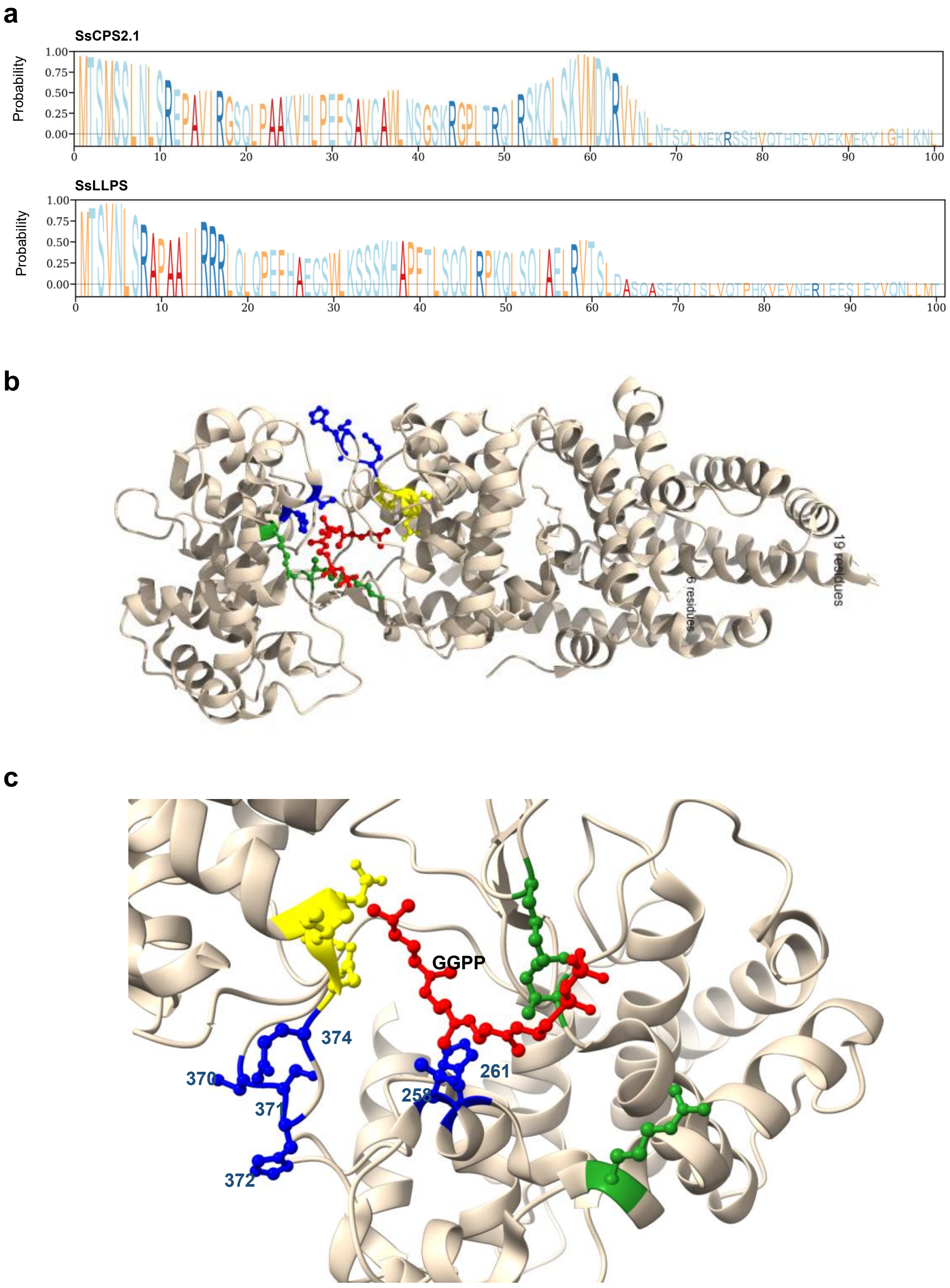
a
SsCPS2.1
Probability
SsLLPS
Probability
b
19 residues
6 residues
c
GGPP
374
370
371
372
258
261

Supplementary Fig. 13. Prediction of protein subcellular localization and 3D structure of the class II diterpene synthase SsCPS2 and active site configuration. (a) SsCPS2.1 and SsLPPS plastid transit peptides. (**b**) Overall ribbon model of the SsCPS2 protein structure, showing the location of the active site. The bound substrate GGPP (geranylgeranyl diphosphate) is shown in red. Residues T258, F261, D370, S371, E372, and C374 targeted by site-directed mutagenesis are highlighted in blue. (**c**) Close-up view of the active site pocket. GGPP is shown in red, surrounded by catalytically important amino acid residues. The DxDD motif is show in yellow.

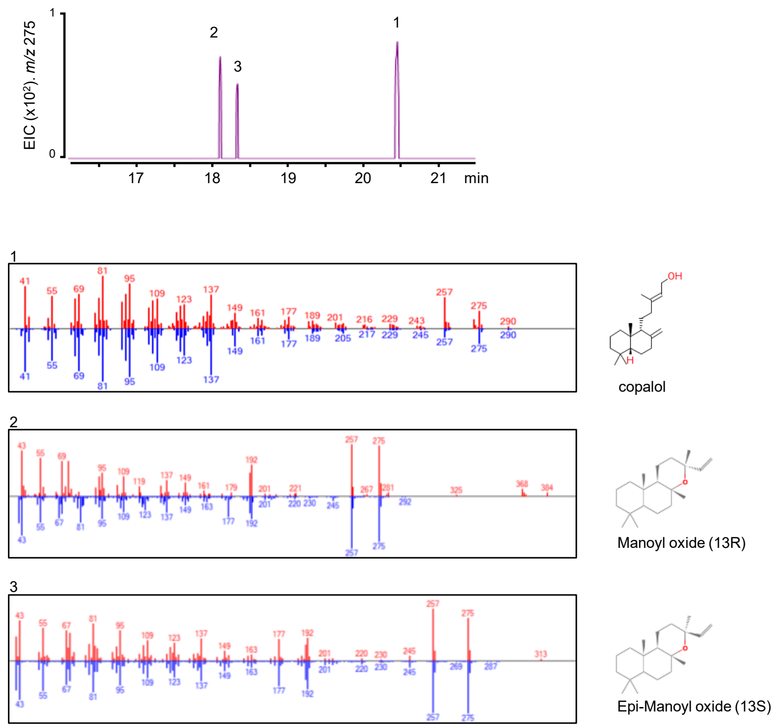

Supplementary Fig. 14. GC–MS analysis of diterpenoid products from enzyme assays. Total ion chromatogram (EIC, *m/z* 275) and mass spectra corresponding to three major peaks identified in the enzymatic product mixture. (1) Copalol, (2) Manoyl oxide (13R)**,** (3) Epi-manoyl oxide (13S)**.** Each spectrum is annotated with major fragment ions, and the corresponding structures are shown on the right. Product identities were confirmed by retention index (RI) and fragmentation pattern comparison with published standards.

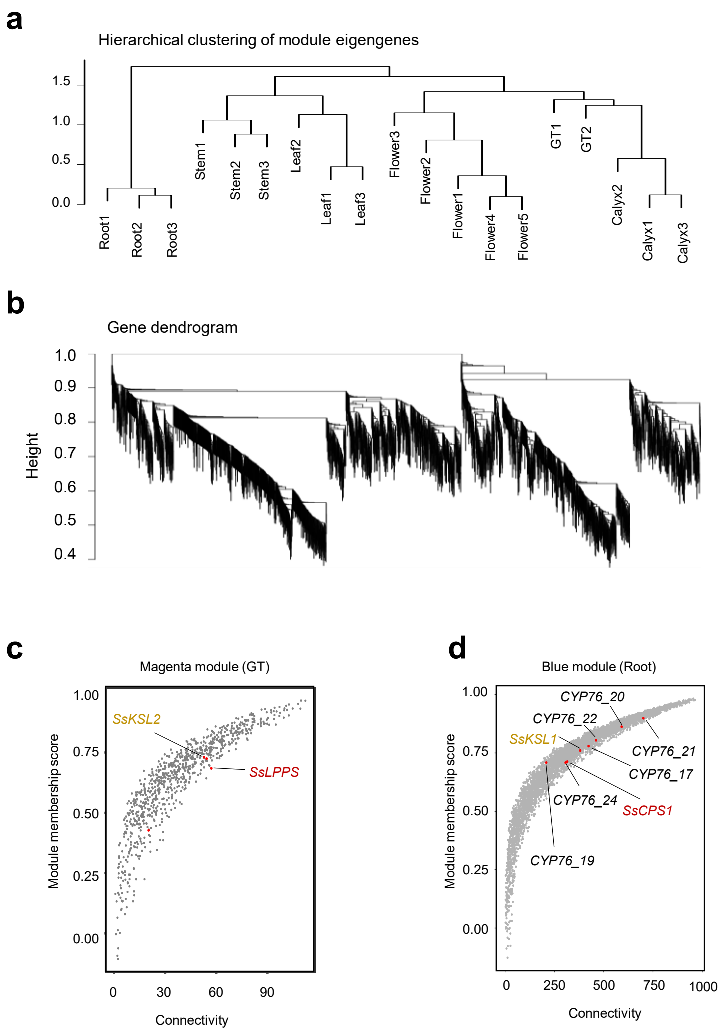

Supplementary Fig. 15. Coexpression analysis across *S. sclarea* tissues. (**a**) Hierarchical clustering of RNA-seq samples used for WGCNA. (**b**) WGCNA gene dendrogram. (**c, d**) Connectivity versus module membership (kME) for genes in the GT-enriched (**c**) and root-enriched (**d**) modules.

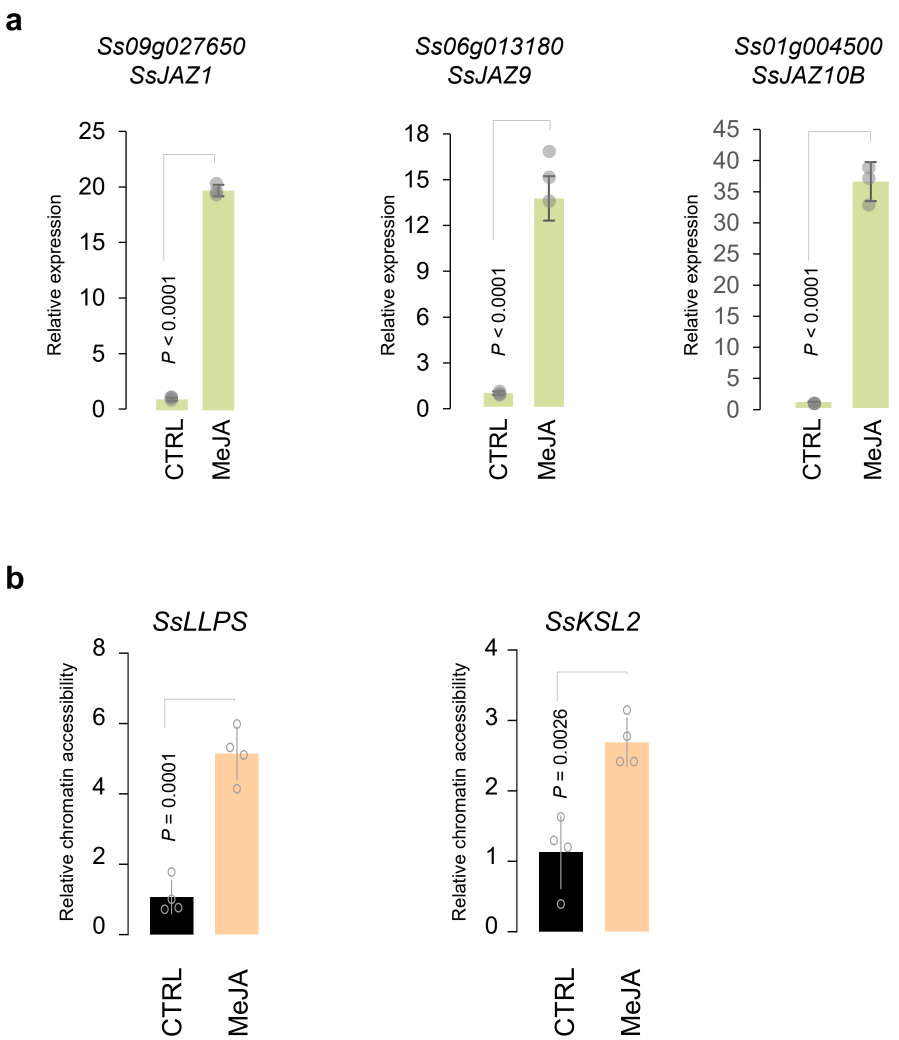

Supplementary Figure 16. MeJA-induced transcription and chromatin accessibility at sclareol biosynthetic gene promoters. (**a**) qRT-PCR analysis of MeJA-responsive JAZ genes (*SsJAZ1*, *SsJAZ9*, *SsJAZ10B*) in control (CTRL) and methyl jasmonate (MeJA) treated tissues. Bars represent mean ± s.d. from three biological replicates. Bars represent the mean ± standard deviation (s.d.) of three biological replicates. (**b**) Chromatin accessibility at the promoters of SsLPPS and SsKSL2 quantified by ATAC-qPCR in control and MeJA-treated tissues. Accessibility values were normalized to a closed intronic genomic region lacking detectable ATAC-seq signal and expressed as relative accessibility ($2^{-\Delta\Delta Ct}$). Bars represent mean ± s.d. from four biological replicates. Statistical significance was assessed using a two-tailed Student's *t*-test. Source data are provided as a Source Data file.

Supplementary Table 1. Statistics of sequencing data used for *S. sclarea* genome assembly and annotation.

	Organ	# of reads	Sequenced bases (Gb)	Average read length (bp)	coverage (X)
Pacbio HiFi	Leaf	2 181 494	27.29	12 520	50.32
ONT	Leaf	4 801 018	44.36	9 240	82.30
Illumina	Leaf	632 997 140	94.94	150	175.08
HiC	Leaf	551 183 597	82.67	150	152.46
ATACseq	Flower	378 883 222	56.83	150	104.80
ATACseq	Root	312 365 354	46.85	150	86.40
ATACseq	Glandular Trichomes	342 423 458	51.36	150	94.71
RNA-seq_ONT	Leaf	2 203 407	2.30	1047.72	
RNA-seq_ONT	Stem	1 924 143	2.03	1055.05	
RNA-seq_ONT	Mature Calyx	1 383 237	1.24	897.81	
RNA-seq_ONT	Flower Bud	2 098 977	2.19	1044.52	
RNA-seq_ONT	Glandular Trichomes	2 652 338	2.25	849.97	

Supplementary Table 2. Statistics of the telomere-to-telomere genome assembly of *S. sclarea*.

Chromosome	Number of contig	Size (bp)
Ss01	1	71 453 059
Ss02	4	61 125 452
Ss03	1	60 691 999
Ss04	2	55 117 540
Ss05	1	53 079 460
Ss06	1	51 333 777
Ss07	1	45 483 023
Ss08	1	42 935 093
Ss09	1	42 186 297
Ss10	1	29 461 910
Ss11	1	29 426 146
Total	15	542 293 756

Supplementary Table 3. *S. sclarea* genome assembly feature comparison.

Genomic feature	This study	Choi *et al.* [1]
Number of chromosome	11	11
Number of contig	15	112
contig N50 (Mb)	45.48	26,29
scaffold N50 (Mb)	54.22	50,47
Total length (bp)	542 293 756	499 032 627
Number of telomeres	20	0
Number of centromeres	11	0
Number on non-TE gene models	32 992	17 202
Complete BUSCOs(%)	99.3	98,7
LAI	23	not determined

Supplementary Table 4. Locations and statistics for the 11 centromeres of the *S. sclarea* genome.

Centromere	Start	End	# of satellite monomers	Length (bp)
Cen01	21 434 741	21 671 014	5 021	236 273
Cen02	54 720 170	55 613 298	18 778	893 128
Cen03	15 967 001	16 611 107	13 576	644 106
Cen04	27 534 832	27 570 684	1379	35 852
Cen05	18 555 451	18 973 621	9 161	418 170
Cen06	46 470 051	48 670 727	12 726	2 200 676
Cen07	22 310 174	23 699 613	23 963	1 389 439
Cen08	39 467 281	41 328 604	31 339	1 861 323
Cen09	1 778 601	2 454 957	23 572	676 356
Cen10	1 017 080	2 317 863	25 450	1 300 783
Cen11	2 478 013	3 764 117	27 346	1 286 104
Average			17 483	994 746
Total			192 311	10 942 210

Supplementary Table 5. Statistics of high-order repeats (HOR) identified in the *S. sclarea* genome.

Chromosome	HOR number	Mean HOR monomers	Mean HOR block (bp)
Chr01	19 233	4.41	701.93
Chr02	198 547	3.75	594.85
Chr03	27 608	4.01	635.34
Chr04	72	5.77	937.95
Chr05	8 502	3.79	600.9
Chr06	287 115	4.7	444.36
Chr07	53 154	3.84	608.89
Chr08	150 692	3.85	609.44
Chr09	230 521	4.74	750.93
Chr10	671 936	3.89	617.75
Chr11	1 047 019	4.7	742.66
Total	2 694 399	4,31	658.64

Supplementary Table 6. Statistics for the 22 telomeres of the *S. sclarea* genome.

Chromosome	Start	End	Monomer	Length	# of repeats
Ss01	1	2 446	TTTAGGG	2 446	349
Ss01	71 447 863	71 453 059	TTTAGGG	5 197	742
Ss02	1	7 032	TTTAGGG	7 032	1005
Ss02	61 121 541	61 125 452	TTTAGGG	3 912	559
Ss03	1	7 206	TTTAGGG	7 206	1029
Ss03	60 688 932	60 691 999	TTTAGGG	3 068	438
Ss04	1	6 493	TTTAGGG	6 493	928
Ss04	55 111 053	55 117 540	TTTAGGG	6 488	927
Ss05	na	na	na		
Ss05	53 074 038	53 079 460	TTTAGGG	5 423	775
Ss06	1	2 821	TTTAGGG	2 821	403
Ss06	51 326 334	51 333 777	TTTAGGG	7 444	1063
Ss07	1	9 937	TTTAGGG	9 937	1420
Ss07	45 473 526	45 483 023	TTTAGGG	9 498	1357
Ss08	1	1 218	TTTAGGG	1 218	174
Ss08	42 929 088	42 935 093	TTTAGGG	6 006	858
Ss09	1	5 592	TTTAGGG	5 592	799
Ss09	42 180 665	42 186 297	TTTAGGG	5 633	805
Ss10	1	7 155	TTTAGGG	7 155	1022
Ss10	29 451 053	29 461 910	TTTAGGG	10 858	1551
Ss11	na	na	na		
Ss11	29 422 183	29 426 146	TTTAGGG	3 964	566

Supplementary Table 7. Distribution and statistics of 5S ribosomal DNA (rDNA) clusters.

Chromosome	Start	End	Length	Element length (bp)	# of repeats
Ss08	40 884 381	40 951 142	66 761	118	194
Ss10	201 857	327 627	125 770	118	370

Supplementary Table 8. BUSCO analysis of the completeness of the *S. sclarea* genome annotation.

	Genome-level		Protein-level	
Description	Number	Percentage	Number	Percentage
Complete BUSCOs (C)	422	99.3	419	98.6
single-copy BUSCOs (S)	412	96.9	410	96.5
duplicated BUSCOs (D)	10	2.4	9	2.1
Fragmented BUSCOs (F)	0	0	5	1.2
Missing BUSCOs (M)	3	0.7	1	0.2
Total BUSCO groups searched	425		425	

lineage : viridiplantae_odb10

Supplementary Table 9. Statistics for gene annotation in the *S. sclarea* genome.

# of gene models	32 992
Totale gene length in genome (bp)	35 157 150
Average gene length (bp)	3019.38
Average CDS length (bp)	1065.63
Average exon length (bp)	365.67
Average intron length (bp)	365.6
Average exon number per gene	3.63

Supplementary Table 10. Statistics for total transposable elements in the *S. sclarea* genome.

Type	Family	#	Length (bp)	Percentage (%)
**Class I: Retrotransposon**				
LTR Retrotransposon				
	Copia	82 511	96 205 402	17.00
	Gypsy	58 963	80 347 497	14.19
	LTR_unknown	62 851	71 014 065	12.55
**Class II: DNA transposon**				
TIR Transposon				
	CACTA	8 772	2 783 120	0.49
	hAT	24 314	9 099 228	1.61
	Mutator	27 527	11 020 455	1.95
	PIF_Harbinger	3 145	1 170 418	0.21
	Tc1_Mariner	61 962	17 656 840	3.12
Non-TIR transposon				
	Helitron	149 087	60 340 581	10.66
**TOTAL**		479 132	349 637 606	61.77

Supplementary Table 11. Statistics of intact transposable elements in the *S. sclarea* genome.

Type	Classification	#
LTR Retrotransposon	Copia	3580
	Gypsy	2475
	unknown	1965
TIR Transposon	MITE	3755
	DTA	1621
	DTM	588
	DTC	222
	DTH	81
	DTT	585
Non-TIR transposon	Helitron	840

Supplementary Table 12. DeepLoc 2.0 signal type prediction for protein subcellular localization.

Protein	Cytoplasm	Nucleus	Extracellular	Cell membrane	Mitochondrion	Plastid	Endoplasmic reticulum	Lysosome/Vacuole	Golgi apparatus	Peroxisome
SsCPS2.1	0.1541	0.1481	0.0276	0.0514	0.2081	0.9497	0.0486	0.1015	0.0778	0.0391
SsLPPS	0.1452	0.1589	0.0211	0.0674	0.1576	0.9453	0.044	0.0917	0.0951	0.0526

Supplementary Table 13. Retention index (RI) values for the three identified peaks and corresponding values from GC–MS libraries and literature.

	**This study**	**Papanikolaou *et al.*[2]**	**Adams [3]**	**FFNSC 2**	**Kuźma *et al.*[4]**	**McCadden *et al.*[5]**	**Milet-Pinheiro *et al.*[6]**
Column	DB-5ms	DB-5	DB-5	SLB-5MS	CP Sil 5 CB	HP-5 MS	HP-5 MS
manoyl oxide (peak 2)	2000	1995	1987	1989	2007		
epi-manoyl oxide (peak 3)	2023	2016	2010	2022	2023		
manool	2057	2050	2057	2062	2070		
sclareol	2223	2212	2223	2225	2231		
n-copalol (peak 1)	2233	2225				2226	
syn-copalol						2266	2262

Supplementary references

1. Choi, S., Kang, Y., Kim, C. Chromosome-level genome assembly of *Salvia sclarea*. *Sci Data* **12**, 14 (2025).
2. Papanikolaou, AS., Papaefthimiou, D., Matekalo, D., Karakousi, CV., Makris, AM., Kanellis, AK. Chemical and transcriptomic analyses of leaf trichomes from *Cistus creticus* subsp. *creticus* reveal the biosynthetic pathways of certain labdane-type diterpenoids and their acetylated forms. *J Exp Bot*. **75**, 3431-3451 (2024).
3. Adams, R. P. Identification of Essential Oil Components by Gas Chromatography/Mass Spectroscopy. 4th ed., Allured Pub. Corp. (2007).
4. Kuźma, L., Kalemba, D., Rózalski, M., Rózalska, B., Wieckowska-Szakiel, M., Krajewska, U., Wysokińska, H. Chemical composition and biological activities of essential oil from *Salvia sclarea* plants regenerated *in vitro*. *Molecules*. **14**, 1438-47 (2009).
5. McCadden, CA., Łomowska-Keehner, DP., Qu, T., Nafie, J., Alsup, TA., Rudolf, JD. Discovery of a plant-like tridomain bifunctional syn-abieta-7,13-diene synthase in Streptomyces. *Org Biomol Chem*. **23**, 9845-9850, (2025).
6. Milet-Pinheiro et al. A semivolatile floral scent marks the shift to a novel pollination system in bromeliads. *Current Biology* **31**, 860-868 (2021)
