# Supplementary material for: Neofunctionalization underlies the evolutionary origin of sclareol biosynthesis in the mint family
Source: Nat Commun. 2026 May 22;17:6745. doi: 10.1038/s41467-026-73637-5 (PMC13385783; doi:10.1038/s41467-026-73637-5)
Supplement: Supplementary file 3 — Description of Additional Supplementary Files [file 41467_2026_73637_MOESM3_ESM.pdf]

Description of Additional Supplementary Files

File Name: Supplementary Data 1
Description: Classification of CYPS450s genes and gene clusters in the Salvia sclarea genome

File Name: Supplementary Data 2
Description: Classification of TPS genes in the Salvia sclarea genome

File Name: Supplementary Data 3
Description: Primers used in this study
