# Supplementary material for: Neofunctionalization underlies the evolutionary origin of sclareol biosynthesis in the mint family
Source: Nat Commun. 2026 May 22;17:6745. doi: 10.1038/s41467-026-73637-5 (PMC13385783; doi:10.1038/s41467-026-73637-5)
Supplement: Supplementary file 2 — Peer Review file [file 41467_2026_73637_MOESM2_ESM.pdf]

nature portfolio

Peer Review File

Neofunctionalization underlies the evolutionary origin of sclareol biosynthesis in the mint family.

Corresponding Author: Dr Adnane Boualem

This file contains all reviewer reports in order by version, followed by all author rebuttals in order by version.

Attachments originally included by the reviewers as part of their assessment can be found at the end of this file.

Version 0:

Reviewer comments:

Reviewer #1

(Remarks to the Author)
This manuscript describes a genome assembly and annotation for Salvia sclarea, which was used to investigate biosynthesis of the diterpene sclareol in the Lamiaceae family.
The assembly is high-quality according to standard metrics. The pangenome comparisons were valuable for showing the expansion of terpene biosynthesis-related genes in S. sclarea relative to other Salvia species. The methyl jasmonate experiment showed clear evidence of transcriptional regulation.

Specific comments:
On page 5, the authors list the telomeric repeat motif as ‘AAACCCT’. This is different from the ‘TTTAGGG’ motif found in most plants. At the same time, in the methods section, the query for the motif search using tidk software is listed as the canonical TTTAGGG. It appears the motif on page 5 is erroneous.

Reviewer #2

(Remarks to the Author)
Focusing on the Lamiaceae species Salvia sclarea, this study deciphers the biosynthetic machinery of the specialized metabolite sclareol. By generating a telomere-to-telomere (T2T) genome assembly and integrating comparative genomics, structural biology, molecular genetics and multi-omic analyses, the authors demonstrate that the sclareol-synthesizing class-II diterpene synthase SsLPPS arose from a recent tandem duplication. Neofunctionalization was achieved through substitution of key amino-acid residues, enabling the enzyme to produce the sclareol direct precursor LPP. SsLPPS further forms a glandular-trichome-specific, co-regulated biosynthetic gene cluster with other diterpenoid genes, illustrating that enzyme innovation coupled with regulatory-network rewiring drives the emergence of a novel plant metabolic pathway and providing both theoretical insights and genetic resources for the sustainable production of high-value terpenoids.
Nevertheless, several shortcomings remain to be addressed:
1. The biosynthetic pathway of sclareol has already been established with a relatively clear framework. In particular, the dual-enzyme route involving SsLPPS and SsSCS from Salvia sclarea was successfully cloned and functionally characterized as early as 2012 through cDNA sequencing (Caniard, A., Zerbe, P., Legrand, S. et al. (2012) Discovery and functional characterization of two diterpene synthases for sclareol biosynthesis in Salvia sclarea (L.) and their relevance for perfume manufacture. BMC Plant Biol 12, 119). Is there any difference in sequence and function between SsCPS2.2 and the reported ScLPPS? Besides, in the following years, this pathway has been reconstructed in various heterologous hosts such as Escherichia coli (Schalk, M., Pastore, L., Mirata, M. A., Khim, S., Schouwey, M., Deguerry, F., Pineda, V., Rocci, L., & Daviet, L. (2012) Toward a biosynthetic route to sclareol and amber odorants. Journal of the American Chemical Society, 134(46), 18900–18903), Saccharomyces cerevisiae (Cao, X., Yu, W., Chen, Y., Yang, S., Zhao, Z. K., Nielsen, J., Luan, H., & Zhou, Y. J. (2023). Engineering yeast for high-level production of diterpenoid sclareol. Metabolic Engineering, 75, 19–28), and more recently in Yarrowia lipolytica with 2.6 gram-per-liter level production (Chen, J., Huang, L., Ye, B. C., & Zhou, Y. (2025). Combinatorial metabolic engineering of Yarrowia lipolytica for high-level production of the plant-derived diterpenoid sclareol. Microbial Cell Factories, 24(1)). Therefore, although the present study employs T2T genome sequencing to explore the evolutionary origin of SsLPPS, its overall novelty is limited, and many of the major conclusions have already been

reported.

2. A chromosome-level genome assembly of Salvia sclarea was published this January (Choi S, Kang Y, Kim C. (2025) Chromosome-level genome assembly of Salvia sclarea. Scientific Data, 12(1): 14). While the authors achieved a gap-free assembly of high quality, they should compare their genome with previously published assemblies (e.g., in Tab. 1).

3. The manuscript only states that the raw NGS data have been deposited in the SRA database under the placeholder PRJNAXXXXX, which does not meet Nature Communications requirement for full data traceability. Please provide the complete SRA accession number(s) and additionally deposit the final genome assembly together with its annotation files; these are essential for reviewers and readers to evaluate the results. Clearly indicate where all other key data sets—genome assembly (fasta), gene annotation (GFF/GTF), ATAC-seq raw reads, and RNA-seq raw reads—have been uploaded, along with their respective accession links or public repository DOIs.

4. Lines 113–115 report the genome annotation results, but only present the genome-level BUSCO score; the BUSCO assessment of the annotated protein set is missing. Please supplement the protein-level BUSCO results to demonstrate the completeness and quality of the gene annotation.

5. Lines 141–144 conclude that core and soft-core gene families are both highly conserved and transcriptionally active. This observation is generic across plants; the authors should clarify whether it has any Salvia sclarea specific evolutionary implications. If not, it should be downgraded to supplementary material. Additionally, Figs 2C and 2D convey essentially the same information and could be merged or one moved to the supplement.

6. Lines 352–378 present the annotation workflow in an illogical order. Please swap the contents of lines 366–370 (protein/gene annotation) with lines 353–364 (repeat annotation) so that repeat masking precedes gene and functional annotation.

7. Fig. 3F presents the in-vitro enzyme assay extracted-ion chromatograms (EIC, m/z 275); however, neither the figure legend nor the main text explicitly assigns peaks 1, 2 and 3 to authenticated standards. Only a general statement “corresponding mass spectra are shown in Fig. S14” is provided. The standards of (+)-copalol, (13R)- and (13S)-manoyl oxides (LPP) should be provided. Alternatively, the known LPPS with the same function should be as the positive control in Fig.3 to confirm the products.

8. The name of SsCPS2.2 or ScLPPS in Fig. 3 should be unified to avoid the confusion and misunderstanding, including in the panels of pathway, phylogenetic tree, sequence alignment, and EIC diagram.

9. The numbers of the compounds should be marked at the bottom of the structure in Fig. 3A.

10. Table 1 lists “Number of contigs” as 23, whereas the Results section states that the final assembly is “542.29 Mb comprised of 15 contigs.” This discrepancy must be checked and corrected to ensure that all key assembly statistics are consistent throughout the manuscript.

11. In the section “Recombinant constructs for protein expression in E. coli”, the authors state that chloroplast transit peptides of SsCPS2.1 and SsLPPS were predicted and truncated, but neither the exact version of the prediction tool (e.g., ChloroP) nor the confidence scores of the predictions are provided. Because different versions can differ markedly in accuracy, these details must be supplied.

12. In the methyl jasmonate (MeJA) treatment experiment, neither the concentration nor the treatment duration of MeJA, nor the details of the control group (e.g., solvent-only), are specified. Moreover, only qRT-PCR was used to monitor expression changes of a subset of genes; chromatin-accessibility data that could reveal MeJA-induced alterations in promoter accessibility were not integrated. Please add these experimental details and incorporate the corresponding ATAC-seq profiles to provide a deeper mechanistic understanding of MeJA-mediated regulation.

13. In the phylogenetic analysis, 17 representative angiosperms were used to construct the Salvia tree, yet the rationale for selecting the out-group (e.g., the non-Lamiaceae Lamiales species Antirrhinum majus) is not stated. Moreover, bootstrap or posterior-probability support values for the key nodes is absent; only the topology is shown in Fig. 2A. Please supply the support values and justify the choice of out-group.

14. Fig. 4B displays the expression patterns of BGC genes across tissues and divides them into two modules—"glandular-trichome/flower-specific" and "root-specific". However, no co-expression network analysis (e.g., WGCNA) was performed to verify that the genes within each module are indeed co-expressed, nor were common cis-regulatory elements in their promoter regions examined. Please add co-expression analyses and promoter motif predictions to substantiate the module assignments.

Reviewer #3

(Remarks to the Author)
In the manuscript, the authors describe the generation of a Salvia sclarea genome assembly, whole genome analyses, and investigations on sclareol biosynthesis. The study discovered that gene duplication and neofunctionalization have enabled this Lamiaceae species to produce sclareol. Both mechanisms are known drivers of chemical diversification in plants

(specialized metabolism). The evolution of sclareol biosynthesis is, thus, another example of a terpenoid biosynthetic pathway in which these mechanisms have played a role.

Major comments to the authors:
Overall, the manuscript lacks focus. For example, a description of whole genome-based data with link to plant specialized metabolism, including a description of the cytochrome P450 superfamily, is followed by sections on sclareol biosynthesis, in which cytochrome P450s are not involved. The S. sclarea genome assembly, combined with a more comprehensive comparative analysis of gene families involved in specialized metabolism across Lamiaceae species, would offer a valuable resource for future investigations into plant specialized metabolism. However, the novelty of the presented results and conclusions drawn regarding sclareol biosynthesis appear to be limited in scope and impact. The discovery of the terpene synthases involved in sclareol biosynthesis and in-depth functional characterization of these enzymes were previously reported (Caniard et al. 2012, doi: 10.1186/1471-2229-12-119). It remains unclear what the authors of the present manuscript hoped to learn from investigating the amino acid substitutions in the terpene synthase. Furthermore, the localization of sclareol biosynthesis in glandular trichomes (GTs) has been previously reported: "…sclareol specifically accumulates in GTs' gland cells in which sclareol biosynthesis genes are strongly expressed" (Chalvin et al. 2021, https://doi.org/10.1038/s41438-021-00640-w). Although not explicitly stated in the previous publication, it stands to reason that the strong, cell-specific co-expression of the genes involved in the same pathway enabled gene discovery.
The discovery of the two inversely regulated gene sets reported in the present manuscript is interesting. However, since the manuscript does not include the identification of a transcription factor binding to the open chromatin region of sclareol biosynthesis genes, the manuscript appears to be more suitable for a genomics-focused journal.

Minor comments to the authors:
- The classification of cytochrome P450s as "clan A" or "non-clan A" is not widely used or accepted. The use of the official terminology (gene family, subfamily, gene number/letter) is recommended.
- Figure 4 lacks a color code.

Version 1:

Reviewer comments:

Reviewer #1

(Remarks to the Author)
My previous comments pertained to minor inconsistencies needing clarification. The authors have satisfied these with their revised version.

Reviewer #2

(Remarks to the Author)
The authors have performed comprehensive and high-quality revisions in response to my core comments. They have supplemented key data (e.g., WGCNA analysis, details of mass spectrometry validation, and phylogenetic support values), resolved data inconsistencies, and refined methodological details, which has significantly improved the scientific rigor and completeness of the study. Several minor points still require clarification:

1.The authors did not provide ATAC-seq data following MeJA treatment, citing that the target tissues are rich in secondary metabolites, highly lignified, and thus difficult for nucleus isolation. While this justification is technically reasonable, and the core goal of the MeJA experiment (verifying the co-expression of BGC genes) has been achieved by qRT-PCR, supplementing schematic diagrams showing changes in chromatin accessibility in the promoter regions of a few key genes (e.g., SsLPPS, SsSCS) after MeJA treatment—even at a qualitative level—would more directly support the conclusion that "MeJA regulates gene expression". Importantly, this gap does not affect the study's core conclusions on enzyme evolution and the origin of regulatory networks. If technically feasible, provide qualitative comparisons of chromatin accessibility for 1–2 core genes (SsLPPS, SsSCS) before and after MeJA treatment (e.g., simplified schematic diagrams of ATAC-seq peak profiles).

2.The authors noted that SsLPPS contains unique amino acid substitutions, but the specific sequence variation sites (e.g., percent identity to ScLPPS and the positions of key substitutions) were not clearly listed in the revised manuscript. Adding this comparative information in a Supplementary Table would more directly validate the sequence specificity of SsLPPS. Supplement a comparison of key sequence variation sites between SsLPPS and ScLPPS (reported in 2012), which can be included in a Supplementary Table

3. In the main Figure 2a, the text annotation "+1598" corresponding to the number of contracted genes for the species S. lycopersicum presents an obvious typesetting misalignment. It is not accurately matched to the branch position of this species, which may easily lead to confusion in data interpretation. The authors are required to correct this figure annotation issue immediately.

4.The Data Availability section of the manuscript does not provide access to the genome assembly sequences (FASTA format) and gene annotation files (GFF/GTF format). These documents are essential for evaluating core quality indicators such as genome assembly continuity and gene annotation accuracy. The authors should supplement the public database

deposition links of the relevant files (e.g., NCBI GenBank, ENA, GigaScience Database) or submit them as supplementary material attachments, to allow peers to conduct a systematic evaluation of the actual genome quality. (This point was raised in the third comment from Reviewer #2, yet the authors still have not provided the corresponding sequence links.)

Reviewer #3

(Remarks to the Author)
Comments to the authors:

Gene duplication and neofunctionalization are already widely recognized as major drivers of the evolution of plant specialized metabolism, and research articles and reviews have provided comprehensive discussions of these processes. Likewise, the biosynthetic pathway for sclareol has been elucidated previously, and the genes involved in sclareol biosynthesis have already been used in biotechnological systems for sclareol production. When viewed in the context of the existing body of work, the findings presented in the manuscript seem limited in impact.

Page 2, last sentence: It is unclear to what extent the findings from the present study form a basis for the sustainable production of high-value plant terpenoids. Rephrase or delete.

Page 6, line 22: Define “condition-dependent” and “low expression”.

Page 6 line 26: A sentence is needed to explain the role of the cytochrome P450 superfamily in chemical diversification to a wider readership.

Page 7, line 32 and page 8, line 1: Introducing two names for one sequence is not helpful. Use either SsKSL3 and SsKSL4 or SsSCS and SsSCS-2 in the manuscript.

Page 8, lines 2 and 3: Use one name for each sequence for clarity. It should be either SsCPS2.2 or SsLPPS. Revise the manuscript accordingly.

Page 17, line 19: Revise “The Pfam domains of genes encoding enzymes…”. Pfam refers to protein families, and domains are the structural and functional building blocks of proteins (not of genes).

Page 18, line 1: Specify the ACTIN gene and add the sequence as supplementary information.

Figures 3i, 3 j and 4d: The light blue color is barely visible in the ATAC-seq chromatin accessibility profiles.

Figure 4: Revise the labeling of the heat map. It is unclear what the color names refer to (e.g. midnightblue (403)).

Page 9, lines 3-13: This paragraph seems out of place. It could be integrated into the following subsection, which reports other ATAC-seq data (see Fig. 4). The graphics in the figures 3 and 4 may need to be rearranged.

Page 10, lines 10-27: Although the authors report MeJA-induced upregulation of sclareol biosynthesis genes, the manuscript does not present corresponding measurements of sclareol accumulation. The data would provide evidence for a correlation between induced gene expression and increased sclareol production and enable more informed conclusions to be drawn.

Version 2:

Reviewer comments:

Reviewer #2

(Remarks to the Author)
The authors have provided clear and thorough responses to all issues raised in the previous round of review, and have made comprehensive and well-structured revisions, effectively addressing the prior shortcomings. The paper is recommended for acceptance without further modifications.

Reviewer #3

(Remarks to the Author)
The revisions made have significantly improved the manuscript’s clarity and scientific rigor. My questions and comments have been addressed.

Open Access This Peer Review File is licensed under a Creative Commons Attribution 4.0 International License, which permits use, sharing, adaptation, distribution and reproduction in any medium or format, as long as you give appropriate credit to the original author(s) and the source, provide a link to the Creative Commons license, and indicate if changes were made.
In cases where reviewers are anonymous, credit should be given to 'Anonymous Referee' and the source.
The images or other third party material in this Peer Review File are included in the article's Creative Commons license, unless indicated otherwise in a credit line to the material. If material is not included in the article's Creative Commons license and your intended use is not permitted by statutory regulation or exceeds the permitted use, you will need to obtain permission directly from the copyright holder.
To view a copy of this license, visit https://creativecommons.org/licenses/by/4.0/

REVIEWER COMMENTS

Reviewer #1 (Remarks to the Author):

This manuscript describes a genome assembly and annotation for Salvia sclarea, which was used to investigate biosynthesis of the diterpene sclareol in the Lamiaceae family.
The assembly is high-quality according to standard metrics. The pangenome comparisons were valuable for showing the expansion of terpene biosynthesis-related genes in S. sclarea relative to other Salvia species. The methyl jasmonate experiment showed clear evidence of transcriptional regulation.

Specific comments:
On page 5, the authors list the telomeric repeat motif as ‘AAACCCT’. This is different from the ‘TTTAGGG’ motif found in most plants. At the same time, in the methods section, the query for the motif search using tidk software is listed as the canonical TTTAGGG. It appears the motif on page 5 is erroneous.

We thank the reviewer for pointing to this. The sequence AAACCCT reported in the Results section corresponds to the reverse-complement of the canonical plant telomeric repeat TTTAGGG, reflecting strand orientation rather than a different telomeric motif.

```
5’-- TTTAGGGTTTAGGGTTTAGGGTTTAGGGTTTAGGG --3’
 3’ ----TCCCAAATCCCAAATCCCAAATCCCAAATCCCAAA --5’
```

The tidk analysis was performed using the canonical TTTAGGG sequence, as stated in the Methods section. For more clarity and to avoid confusion, we have revised the Results and Supplementary Table 6 text to consistently refer to the canonical motif throughout the manuscript.

Reviewer #2 (Remarks to the Author):

Focusing on the Lamiaceae species Salvia sclarea, this study deciphers the biosynthetic machinery of the specialized metabolite sclareol. By generating a telomere-to-telomere (T2T) genome assembly and integrating comparative genomics, structural biology, molecular genetics and multi-omic analyses, the authors demonstrate that the sclareol-synthesizing class-II diterpene synthase SsLPPS arose from a recent tandem duplication. Neofunctionalization was achieved through substitution of key amino-acid residues, enabling the enzyme to produce the sclareol direct precursor LPP. SsLPPS further forms a glandular-trichome-specific, co-regulated biosynthetic gene cluster with other diterpenoid genes, illustrating that enzyme innovation coupled with regulatory-network rewiring drives the emergence of a novel plant metabolic pathway and providing both theoretical insights and genetic resources for the sustainable production of high-value terpenoids.

Nevertheless, several shortcomings remain to be addressed:
1. The biosynthetic pathway of sclareol has already been established with a relatively clear framework. In particular, the dual-enzyme route involving SsLPPS and SsSCS from Salvia sclarea was successfully cloned and functionally characterized as early as 2012 through cDNA sequencing (*Caniard, A., et al. 2012*). Is there any difference in sequence and function between SsCPS2.2 and the reported ScLPPS? Besides, in the following years, this pathway has been reconstructed in various heterologous hosts such as Escherichia coli (*Schalk, M., et al., (2012),* Saccharomyces cerevisiae (*Cao, X., et al. 2023*), and more recently in Yarrowia lipolytica with 2.6 gram-per-liter level production (*Chen, J., et al., 2025*). Therefore, although the present study employs T2T genome sequencing to explore the evolutionary origin of SsLPPS, its overall novelty is limited, and many of the major conclusions have already been reported.

We thank the reviewer for their careful evaluation and recognition of the quality of the genome assembly and analyses, and we appreciate the opportunity to clarify the study's scope and novelty.

We agree that Caniard et al. (2012) made a valuable contribution by identifying *in vitro* activities consistent with a two-enzyme route to sclareol formation. However, those findings do not address, nor do they attempt to address, the central questions that our study resolves:

(i) how the sclareol-producing activity evolved,
(ii) which genetic events created this function, and
(iii) which specific amino-acid changes reprogrammed the enzyme active site to generate the LPP intermediate.

These questions have remained unanswered for more than a decade despite pathway reconstruction in microbial systems.

1. Sequence and functional distinctiveness of SsLPPS (SsCPS2.2).
In Caniard et al. (2012), the enzyme reported as "ScLPPS" is a diterpene synthase isolated from a cDNA library, but its genomic context, copy number, and evolutionary origin were unknown. Our T2T genome resolves this locus for the first time and shows that *S. sclarea* contains **two adjacent class II diterpene synthases**, SsCPS2.1 and SsCPS2.2 (SsLPPS), arising from a **recent, lineage-specific tandem duplication** that is absent from related *Salvia* species. Sequence comparison, structural modeling, and mutagenesis in our study demonstrate that **SsCPS2.2 possesses unique amino-acid substitutions that causally underlie LPP formation**, distinguishing it from both SsCPS2.1 and the enzyme initially reported from cDNA.
Thus, the enzyme characterized in 2012 corresponds functionally to SsLPPS but its **origin, divergence, and causal substitutions** were unknown and are elucidated only here.

2. Prior pathway reconstructions do not address evolutionary mechanism.
Microbial production studies (Schalk 2012; Cao 2023; Chen 2025) confirm that heterologous expression can generate sclareol, but they do not identify the **duplication event** that created SsLPPS, the **ancestral**

state of the enzyme, the **amino-acid determinants** that reprogrammed catalytic specificity, the **regulatory organization** of the pathway, or its **integration into a jasmonate-responsive gene cluster**. Our study provides the first **evolutionary, mechanistic, and regulatory dissection** of the pathway.

3. Substantial conceptual advances introduced in this study.

We respectfully emphasize that the novelty of our work lies not in re-identifying pathway enzymes, but in revealing *how* the pathway originated and is regulated. Specifically, our work shows:

1. **A recent, lineage-specific tandem duplication** generated the precursor of SsLPPS.
2. **Few amino-acid substitutions** are sufficient to convert the ancestral CPS into a neofunctionalized LPP synthase (proven by mutagenesis and structural modeling).
3. The sclareol pathway resides in a **chromatin-regulated, trichome-specific, jasmonate-responsive biosynthetic gene cluster**—a regulatory architecture not previously described.
4. The T2T genome provides **complete resolution** of the cluster, enabling insights not possible from earlier, fragmented assemblies or cDNA-based work.

These findings **could not be inferred** from prior biochemical studies or heterologous reconstructions.

Thanks to the reviewer that raised this question, now we explicitly contrast our findings with earlier work to clarify the conceptual novelty of our manuscript. Our work describes the **genomic origin, evolutionary trajectory, causal mutations, and the epigenetic regulation** underlying this pathway.

2. A chromosome-level genome assembly of Salvia sclarea was published this January (Choi S, Kang Y, Kim C. (2025) Chromosome-level genome assembly of Salvia sclarea. Scientific Data, 12(1): 14). While the authors achieved a gap-free assembly of high quality, they should compare their genome with previously published assemblies (e.g., in Tab. 1).

We thank the reviewer for raising this point. We have now included a detailed comparison between our assembly and the *S. sclarea* genome published by Choi et al. (2025), both in Supplementary Table 3 and in the Results section.

While the assembly of Choi et al. meets the technical criteria of *Scientific Data*, it remains highly fragmented (112 contigs across 72 scaffolds), does not resolve telomeres or centromeres, and recovers only 17,202 non-TE gene models. As is standard for *Scientific Data*, the article presents a data descriptor without assessment of conceptual novelty, biological interpretation, or mechanistic insight.

In contrast, our telomere-to-telomere genome assembly shows substantially higher contiguity (15 contigs; contig N50 = 45.48 Mb vs. 26.29 Mb), resolves all **20 telomeres** and all **11 centromeres**, recovers nearly twice as many non-TE coding genes (32,992), and achieves a higher BUSCO completeness (99.3%). Our assembly is ~43 Mb longer, consistent with full recovery of repetitive and structurally complex regions, and has an LAI of 23 (not reported for Choi et al.).

These improvements were essential for the mechanistic and evolutionary analyses that form the core of our study. Specifically, the T2T assembly enabled us to:

(i) resolve the **recent tandem duplication** at the diterpene synthase locus;
(ii) identify the **amino-acid substitutions** underlying enzyme neofunctionalization; and
(iii) reconstruct the **trichome-specific, jasmonate-responsive** diterpenoid biosynthetic gene cluster.

These biological and mechanistic insights extend well beyond the scope of a data descriptor and were not addressed by Choi et al. (2025).

3. The manuscript only states that the raw NGS data have been deposited in the SRA database under the placeholder PRJNAXXXXX, which does not meet Nature Communications requirement for full data traceability. Please provide the complete SRA accession number(s) and additionally deposit the final genome assembly together with its annotation files; these are essential for reviewers and readers to evaluate the results. Clearly indicate where all other key data sets—genome assembly (fasta), gene annotation (GFF/GTF), ATAC-seq raw reads, and RNA-seq raw reads—have been uploaded, along with their respective accession links or public repository DOIs.

All sequencing data have now been deposited in public repositories. Complete accession numbers for WGS, RNA-seq, ATAC-seq, and bisulfite sequencing are provided in the Data Availability section.

4. Lines 113–115 report the genome annotation results, but only present the genome-level BUSCO score; the BUSCO assessment of the annotated protein set is missing. Please supplement the protein-level BUSCO results to demonstrate the completeness and quality of the gene annotation.

We thank the reviewer for this suggestion. We have now included protein-level BUSCO assessments in addition to genome-level scores. These results are now reported in the revised Supplementary Table 8.

5. Lines 141–144 conclude that core and soft-core gene families are both highly conserved and transcriptionally active. This observation is generic across plants; the authors should clarify whether it has any Salvia sclarea specific evolutionary implications. If not, it should be downgraded to supplementary material. Additionally, Figs 2C and 2D convey essentially the same information and could be merged or one moved to the supplement.

We thank the reviewer for this insightful comment. We agree that conservation and broad expression of core and soft-core gene families is a generic feature of plant pan-genomes. To clarify the *S. sclarea*-specific relevance of this analysis, we now explicitly link these observations to the emergence of lineage-specific innovations. In line with the reviewer's suggestion, we have added the following sentence at the end of the paragraph:

"Core genes exhibited broader expression across tissues and higher functional annotation, whereas specific genes showed condition-dependent or low expression, reflecting a pattern that is generic across plant pan-genomes (Fig. 2d, e). In *S. sclarea*, these conserved and broadly expressed core and soft-core families provide a stable genomic background from which lineage-specific expansions in specialized metabolism, particularly TPS and CYP450 families, have diversified."

We also appreciate the suggestion regarding redundancy between Figs. 2c and 2d. In the revised manuscript, we moved the detailed breakdown of cluster categories to the Supplementary Figure 9b.

These changes help sharpen the focus of the main text on *S. sclarea*–specific genomic features that directly support the evolutionary and mechanistic findings presented in the study.

6. Lines 352–378 present the annotation workflow in an illogical order. Please swap the contents of lines 366–370 (protein/gene annotation) with lines 353–364 (repeat annotation) so that repeat masking precedes gene and functional annotation.
As suggested by the reviewer, we have reorganized the Methods section so that repeat annotation precedes gene and functional annotation**,** consistent with standard genome annotation workflows.

7. Fig. 3F presents the in-vitro enzyme assay extracted-ion chromatograms (EIC, m/z 275); however, neither the figure legend nor the main text explicitly assigns peaks 1, 2 and 3 to authenticated standards. Only a general statement "corresponding mass spectra are shown in Fig. S14" is provided. The standards of (+)-copalol, (13R)- and (13S)-manoyl oxides (LPP) should be provided. Alternatively, the known LPPS with the same function should be as the positive control in Fig.3 to confirm the products.

We thank the reviewer for this comment and agree that the assignments of peaks 1, 2, and 3 in Fig. 3F required clearer justification. In the revised manuscript, we now explicitly assign each peak in both the main text and the figure legend, and provide the analytical criteria used for identification in the methods. As we did not have access to purified (+)-copalol or (13R)/(13S)-manoyl oxide standards, we followed the commonly accepted identification procedure for diterpenes based on: (i) retention index (RI) comparison, (ii) EI mass spectral matching against validated libraries (NIST and FFNSC2), and (iii) comparison with published reference spectra and RI values (now included in Supplementary Table 15).

These additions make the peak assignments transparent and reproducible. The revised legend now states:

- **Peak 1**: copalol
- **Peak 2**: manoyl oxide (13R)
- **Peak 3**: Epi-manoyl oxide (13S)

The corresponding EI mass spectra and RI comparisons are now shown in Supplementary Fig. 14 and detailed in Supplementary Table 15.

We also note that previous studies of LPPS/CPS enzymes have used the same identification strategy (reference n°6), and that the detection of the expected manoyl oxides and copalol products is fully consistent with the known enzymatic function of copalyl diphosphate synthase–like enzymes.

8. The name of SsCPS2.2 or ScLPPS in Fig. 3 should be unified to avoid the confusion and misunderstanding, including in the panels of pathway, phylogenetic tree, sequence alignment, and EIC diagram.
We thank the reviewer for pointing out this inconsistency. We have now fully unified the gene nomenclature across the entire manuscript, including all figures (biosynthetic pathway, phylogenetic tree, sequence alignment, EIC chromatograms) and supplementary materials. All references to this enzyme now consistently use the name SsLPPS to prevent confusion and ensure clarity.

9. The numbers of the compounds should be marked at the bottom of the structure in Fig. 3A.
We thank the reviewer for this suggestion. The compound numbers have now been added beneath each chemical structure in the revised Fig. 3a.

10. Table 1 lists "Number of contigs" as 23, whereas the Results section states that the final assembly is "542.29 Mb comprised of 15 contigs." This discrepancy must be checked and corrected to ensure that all key assembly statistics are consistent throughout the manuscript.

We thank the reviewer for noticing this discrepancy. The correct number of contigs in our final genome assembly is 15, and we have now updated Table 1 and all relevant sections of the manuscript accordingly. This low contig number reflects the high continuity and completeness of our telomere-to-telomere assembly, further demonstrating its superior quality.

11. In the section "Recombinant constructs for protein expression in E. coli", the authors state that chloroplast transit peptides of SsCPS2.1 and SsLPPS were predicted and truncated, but neither the exact

version of the prediction tool (e.g., ChloroP) nor the confidence scores of the predictions are provided. Because different versions can differ markedly in accuracy, these details must be supplied.

As requested by the reviewer, we now specify the prediction tool, version, and confidence scores used for chloroplast transit peptide identification. Specifically, we used DeepLoc 2.0 to predict subcellular localization and to estimate the probability of plastid targeting for SsCPS2.1 and SsLPPS. The predicted plastid transit peptide probabilities and confidence scores have been added to Supplementary Fig. 13, and the Methods section has been updated accordingly to reflect these details.

12. In the methyl jasmonate (MeJA) treatment experiment, neither the concentration nor the treatment duration of MeJA, nor the details of the control group (e.g., solvent-only), are specified. Moreover, only qRT-PCR was used to monitor expression changes of a subset of genes; chromatin-accessibility data that could reveal MeJA-induced alterations in promoter accessibility were not integrated. Please add these experimental details and incorporate the corresponding ATAC-seq profiles to provide a deeper mechanistic understanding of MeJA-mediated regulation.

We thank the reviewer for this important comment. We have now added full experimental details for the MeJA treatment, including the MeJA concentration (1 mM), the presence of 0.1% (v/v) Tween-20 as solvent, the total application volume (50 mL per plant), the use of solvent-only controls, and the treatment duration (6 h). These details are now provided in the Methods section.

Regarding the suggestion to include ATAC-seq data from MeJA-treated tissues, we appreciate the reviewer’s insight. Despite we have a long experience in ATAC-seq I our team, we found generating ATAC-seq data from *S. sclarea* plants treated with MeJA is technically challenging: the tissues are rich in secondary metabolites, highly sclerified, and not well suited for nuclei isolation after chemical treatments.

The goal of the MeJA treatment in our study was to **confirm the coordinated transcriptional regulation of the genes within the biosynthetic gene cluster**. Our qRT-PCR results show that the cluster behaves as a coherent transcriptional unit across tissues and under MeJA treatment, which is the level of evidence required to support our conclusions.

As the central message of our study is the **evolutionary and mechanistic origin of the neofunctionalized SsLPPS enzyme and the structural organization of the diterpenoid biosynthetic gene cluster**, we believe that chromatin-level MeJA responses are undoubtedly interesting, but fall outside the main scope of the manuscript.

For more clarity and to take in consideration the reviewer comment, we have clarified the purpose of the MeJA experiment in the revised text and now explicitly state that its role is to demonstrate coordinated expression within the gene cluster.

13. In the phylogenetic analysis, 17 representative angiosperms were used to construct the Salvia tree, yet the rationale for selecting the out-group (e.g., the non-Lamiaceae Lamiales species Antirrhinum majus) is not stated. Moreover, bootstrap or posterior-probability support values for the key nodes is absent; only the topology is shown in Fig. 2A. Please supply the support values and justify the choice of out-group.
We thank the reviewer for raising this question. In phylogenomic species-tree reconstruction, the concept of a single explicit *out-group* is not required in the same way as for single-gene phylogenies. Our species tree was inferred from a concatenated set of single-copy orthologs across 17 angiosperms and subsequently time-calibrated using multiple independent fossil-based calibration points. In such

phylogenomic frameworks, the tree is rooted through the calibration constraints rather than through the inclusion of a designated out-group.
Antirrhinum majus (Lamiales, Plantaginaceae) was included not as a formal out-group, but as a representative non-Lamiaceae Lamiales species to provide broader phylogenetic context within the Lamiales clade. Its placement helps stabilize orthogroup inference and divergence-time estimation but does not act as the sole root-defining species.

To comply with the reviewer, we have now added bootstrap support values (1,000 replicates) for all major nodes. The updated version of Fig. 2a displays these support values directly on the tree.

14. Fig. 4B displays the expression patterns of BGC genes across tissues and divides them into two modules—"glandular-trichome/flower-specific" and "root-specific". However, no co-expression network analysis (e.g., WGCNA) was performed to verify that the genes within each module are indeed co-expressed, nor were common cis-regulatory elements in their promoter regions examined. Please add co-expression analyses and promoter motif predictions to substantiate the module assignments.

We thank the reviewer for this insightful suggestion. In response, we performed a weighted gene co-expression network analysis (WGCNA) using transcriptomic data from six *S. sclarea* tissues. This analysis robustly recovered two transcriptional modules that correspond to the GT-enriched and root-enriched groups originally identified by hierarchical clustering (now shown in Fig. 4B and Supplementary Fig. 15). The GT module includes SsLPPS and SsKSL2, whereas the root module contains SsCPS1, SsKSL1, and several CYP76 genes, confirming that the two groups of BGC genes are indeed co-expressed within their respective tissues.

Regarding cis-regulatory features, our chromatin-accessibility maps show that promoters of GT-expressed genes exhibit greater accessibility in glandular trichomes, whereas promoters of root-expressed genes display stronger accessibility signals in roots (Figs. 3j and 4d). These patterns support cis-regulatory divergence between the two transcriptional modules.

Together, the co-expression analysis and chromatin-accessibility profiles substantiate the module assignments and reinforce the conclusion that the diterpene BGC is partitioned into two physiologically distinct and independently regulated units. Although promoter-motif discovery would provide an additional layer of regulatory information, such an analysis lies beyond the scope of the present study and would not alter our conclusions regarding coordinated expression within the cluster.

Reviewer #3 (Remarks to the Author):

In the manuscript, the authors describe the generation of a Salvia sclarea genome assembly, whole genome analyses, and investigations on sclareol biosynthesis. The study discovered that gene duplication and neofunctionalization have enabled this Lamiaceae species to produce sclareol. Both mechanisms are known drivers of chemical diversification in plants (specialized metabolism). The evolution of sclareol biosynthesis is, thus, another example of a terpenoid biosynthetic pathway in which these mechanisms have played a role.

We thank Reviewer 3 for the thoughtful and constructive evaluation of our work. We sincerely appreciate the recognition that gene duplication and neofunctionalization are key drivers of metabolic diversification in plants and that our discovery of the two inversely regulated modules is scientifically interesting. Below, we respond to each point and clarify the novelty, focus, and contribution of our study.

Major comments to the authors:
Overall, the manuscript lacks focus. For example, a description of whole genome-based data with link to plant specialized metabolism, including a description of the cytochrome P450 superfamily, is followed by sections on sclareol biosynthesis, in which cytochrome P450s are not involved. The S. sclarea genome assembly, combined with a more comprehensive comparative analysis of gene families involved in specialized metabolism across Lamiaceae species, would offer a valuable resource for future investigations into plant specialized metabolism.

We thank the reviewer for the constrictive suggestion. In the revised manuscript, we have improved the narrative flow by explicitly linking each genomic section to the biological question motivating the study.

In our initial attention of the genome-wide analysis, of the CYP450 superfamily, aimed to contextualize **the evolutionary dynamics of specialized metabolism across Salvia,** and highlight that **S. sclarea shows lineage-specific expansion of specialized metabolic gene families**. This provides an evolutionary framework within which sclareol biosynthesis emerged.

To clarify the objective of genome-wide analysis, of the CYP450 superfamily, we rephrased the relevant section and clarified its purpose in the Introduction, Results and Discussion.

However, the novelty of the presented results and conclusions drawn regarding sclareol biosynthesis appear to be limited in scope and impact. The discovery of the terpene synthases involved in sclareol biosynthesis and in-depth functional characterization of these enzymes were previously reported (Caniard et al. 2012, doi: 10.1186/1471-2229-12-119).

We appreciate this important point. The prior study by Caniard et al. (2012) identified two TPSs capable of producing intermediates in the pathway but **did not address the following questions:**

- **What is the evolutionary origin** of these TPS enzymes?
- **What are the causal amino acid substitutions** responsible for neofunctionalization?
- **How the mechanistic shift in catalytic specificity occurred?**
- **What are the regulatory architecture** that enabled trichome-specific expression?

<u>New results unique to our work</u>:

1. **Identification of a recent, lineage-specific tandem duplication (SsCPS2.1 → SsLPPS)** that is absent from related Salvia species.

2. **Causal demonstration of enzymatic neofunctionalization**, showing that only six amino acid substitutions are sufficient to reprogram substrate positioning and specificity.
3. **Structural modeling + site-directed mutagenesis** revealing how active-site remodeling reshaped the catalytic outcome.
4. **Discovery of a trichome-specific, chromatin-accessible, jasmonate-responsive diterpene biosynthetic gene cluster (BGC)** containing the neofunctionalized gene and its partner TPS.
5. **Integration of T2T genome assembly, ATAC-seq, phylogenomics, and comparative synteny** to reconstruct the evolutionary history and regulatory specialization of the pathway.

Thus, while the existence of two TPSs was previously known, **their evolutionary origin, functional divergence, structural changes, and regulatory integration had never been resolved before**.

We hope in the new submitted revision, by making our work more focused, we have clarified its novelty.

It remains unclear what the authors of the present manuscript hoped to learn from investigating the amino acid substitutions in the terpene synthase.

One fundamental evolutionary question is how **a new enzyme catalytic activity emerges?**

Through the genomic and functional characterization of CPS and LPPS in scariol producing and non producing species, we identified a small set of amino acid changes can switch a CPS into an LPPS, Through his work we provide:

- **direct evidence for how new metabolic functions originates**,
- **molecular dissection of the neofunctionalization process**, and
- an **experimentally validated model for the origins of chemical novelty**.

This is a major conceptual contribution that goes far beyond pathway characterization.

Furthermore, the localization of sclareol biosynthesis in glandular trichomes (GTs) has been previously reported: "…sclareol specifically accumulates in GTs' gland cells in which sclareol biosynthesis genes are strongly expressed" (Chalvin et al. 2021, https://doi.org/10.1038/s41438-021-00640-w). Although not explicitly stated in the previous publication, it stands to reason that the strong, cell-specific co-expression of the genes involved in the same pathway enabled gene discovery.

Indeed, in our previous work (Chalvin et al, 2021) we reported trichome localization of sclareol accumulation. In the present study, our contribution is to show **how this tissue specificity is encoded at the regulatory and chromatin levels**.

Specifically, our study newly demonstrates that:

- **Promoters of SsLPPS and SsSCS exhibit strong chromatin accessibility specifically in trichomes**,
- The genes belong to a **coherent transcriptional module**,
- They are embedded within a **compact diterpene BGC**,
- The BGC is **coordinately regulated by jasmonate**, with GT and root modules showing **opposite hormonal responses**,
- This regulatory configuration evolved in parallel with enzyme neofunctionalization.

This integrated genomic–regulatory perspective was not available previously.

The discovery of the two inversely regulated gene sets reported in the present manuscript is interesting. However, since the manuscript does not include the identification of a transcription factor binding to the open chromatin region of sclareol biosynthesis genes, the manuscript appears to be more suitable for a genomics-focused journal.

We have revised our manuscript so that the biological significance is clearer. While our study uses genomic approaches, we have (i) elucidated, how a new metabolic pathway emerges, (ii) discovered the causal mutations enabling new enzymatic activities; (iii) demonstrated how gene duplication and neofunctionalization led to the diversification of specialized metabolite, central to plant ecology and industry.

These are mechanistic insights that aligne closely with the biological focus of *Nature Communications*. Nonetheless, we genuinely appreciate the reviewer's perspective and have improved clarity and focus accordingly.

Minor comments to the authors:
- The classification of cytochrome P450s as "clan A" or "non-clan A" is not widely used or accepted. The use of the official terminology (gene family, subfamily, gene number/letter) is recommended.

We thank the reviewer for these helpful suggestions. Cytochrome P450 nomenclature has been updated to follow standard conventions, and figure legends and color codes have been clarified.

- Figure 4 lacks a color code.

The color code has been added in the revised figure 4.

REVIEWER COMMENTS

Reviewer #1 (Remarks to the Author):

My previous comments pertained to minor inconsistencies needing clarification. The authors have satisfied these with their revised version.

Reviewer #2 (Remarks to the Author):

The authors have performed comprehensive and high-quality revisions in response to my core comments. They have supplemented key data (e.g., WGCNA analysis, details of mass spectrometry validation, and phylogenetic support values), resolved data inconsistencies, and refined methodological details, which has significantly improved the scientific rigor and completeness of the study. Several minor points still require clarification:

1.The authors did not provide ATAC-seq data following MeJA treatment, citing that the target tissues are rich in secondary metabolites, highly lignified, and thus difficult for nucleus isolation. While this justification is technically reasonable, and the core goal of the MeJA experiment (verifying the co-expression of BGC genes) has been achieved by qRT-PCR, supplementing schematic diagrams showing changes in chromatin accessibility in the promoter regions of a few key genes (e.g., SsLPPS, SsSCS) after MeJA treatment—even at a qualitative level—would more directly support the conclusion that "MeJA regulates gene expression". Importantly, this gap does not affect the study's core conclusions on enzyme evolution and the origin of regulatory networks. If technically feasible, provide qualitative comparisons of chromatin accessibility for 1–2 core genes (SsLPPS, SsSCS) before and after MeJA treatment (e.g., simplified schematic diagrams of ATAC-seq peak profiles).

We thank the reviewer for this helpful suggestion. As noted in our previous response, generating genome-wide ATAC-seq libraries from MeJA-treated *S. sclarea* tissues proved technically challenging because these tissues are rich in secondary metabolites and highly sclerified, which complicates the isolation of sufficient numbers of high-quality nuclei required for genome-wide chromatin profiling.

To further strengthen our conclusions, after the submission of the first revision we undertook targeted ATAC-qPCR experiments to directly assess chromatin accessibility at key loci of the chromosome 8 diterpene biosynthetic gene cluster. ATAC-qPCR requires substantially fewer nuclei and does not depend on genome-wide library complexity, making it suitable for targeted validation even when full ATAC-seq profiling is difficult to obtain.

Using this approach, we quantified chromatin accessibility at the promoters of SsLPPS and SsKSL2 in control and MeJA-treated tissues. These experiments revealed a significant increase in chromatin accessibility following MeJA treatment, consistent with transcriptional activation of the biosynthetic gene cluster.

These new results are presented in Supplementary Fig. 16, and the corresponding experimental details have been added to the Methods section, with the primers used listed in Supplementary Table 14. Together, these data provide direct evidence that jasmonate signaling is associated with increased promoter accessibility at key genes of the sclareol biosynthetic module, supporting the regulatory model proposed in the manuscript.

2.The authors noted that SsLPPS contains unique amino acid substitutions, but the specific sequence variation sites (e.g., percent identity to ScLPPS and the positions of key substitutions) were not clearly listed in the revised manuscript. Adding this comparative information in a Supplementary Table would more directly validate the sequence specificity of SsLPPS. Supplement a comparison of key sequence variation sites between SsLPPS and ScLPPS (reported in 2012), which can be included in a Supplementary Table

We thank the reviewer for this suggestion. As explained in our response during the first revision, the enzyme reported as ScLPPS in Caniard et al. (2012) was identified from a cDNA library, and therefore its genomic context, copy number, and evolutionary origin were unknown at that time. Our telomere-to-telomere genome assembly resolves this locus and demonstrates that *S. sclarea* contains two adjacent class II diterpene synthases, SsCPS2.1 and SsCPS2.2 (SsLPPS), which arose from a recent lineage-specific tandem duplication that is absent from related *Salvia* species.

The SsLPPS sequence identified in this study corresponds to the previously reported ScLPPS enzyme described by Caniard et al. (2012). A direct comparison of the two protein sequences revealed that they are nearly identical, differing by only three amino acid substitutions. To clarify this point, we have now highlighted these residues in blue in Supplementary Fig. 12, which explicitly shows the positions of these substitutions within the multiple sequence alignment.

This comparison confirms that the enzyme characterized here corresponds to the previously described LPPS activity, while our work provides additional insight into its evolutionary origin, structural determinants of specificity, and genomic organization within a biosynthetic gene cluster. To further clarify this point for readers, we explicitly cite Caniard et al. (2012) the first time SsLPPS is introduced in the Results section (page 8, line 3), acknowledging the prior biochemical characterization of this enzyme.

3. In the main Figure 2a, the text annotation "+1598" corresponding to the number of contracted genes for the species S. lycopersicum presents an obvious typesetting misalignment. It is not accurately matched to the branch position of this species, which may easily lead to confusion in data interpretation. The authors are required to correct this figure annotation issue immediately.

We thank the reviewer for pointing out this formatting issue. The misalignment of the annotation corresponding to *S. lycopersicum* occurred during the generation of the PDF version of the figure and was not present in the original figure file. This issue has now been corrected in the revised Fig. 2a, where the annotation is properly aligned with the corresponding branch to avoid any potential confusion in data interpretation. During this revision, we also carefully checked all figure annotations to ensure consistent alignment and formatting throughout the manuscript.

4.The Data Availability section of the manuscript does not provide access to the genome assembly sequences (FASTA format) and gene annotation files (GFF/GTF format). These documents are essential for evaluating core quality indicators such as genome assembly continuity and gene annotation accuracy. The authors should supplement the public database deposition links of the relevant files (e.g., NCBI GenBank, ENA, GigaScience Database) or submit them as supplementary material attachments, to allow peers to conduct a systematic evaluation of the actual genome quality. (This point was raised in the third comment from Reviewer #2, yet the authors still have not provided the corresponding sequence links.)

We thank the reviewer for emphasizing the importance of making the genome assembly and annotation files publicly available and apologize for the oversight in the previous revision. The *Salvia sclarea*

genome assembly (FASTA format) and the corresponding gene annotation files (GFF3 format) have now been deposited under the NCBI GenBank BioProject PRJNA1417963.

To further facilitate independent evaluation of genome assembly continuity and gene annotation quality, the genome assembly and associated annotation files have also been deposited in the Recherche Data Gouv repository and are accessible at the following URL: https://dora.ips2.universite-paris-saclay.fr/previewurl.xhtml?token=c496c266-b34a-4362-8849-0ef09ddce1e3

The Data Availability section of the manuscript has been updated accordingly.

Reviewer #3 (Remarks to the Author):

Comments to the authors:

Gene duplication and neofunctionalization are already widely recognized as major drivers of the evolution of plant specialized metabolism, and research articles and reviews have provided comprehensive discussions of these processes. Likewise, the biosynthetic pathway for sclareol has been elucidated previously, and the genes involved in sclareol biosynthesis have already been used in biotechnological systems for sclareol production. When viewed in the context of the existing body of work, the findings presented in the manuscript seem limited in impact.

We thank the reviewer for raising this point and for recognizing that gene duplication and neofunctionalization are well-established mechanisms in the evolution of plant specialized metabolism. Our goal in this study was not to re-establish these general principles, but rather to provide **a** mechanistic and evolutionary dissection of how such a process occurred in a specific metabolic innovation.

Previous work identified the enzymes involved in sclareol biosynthesis through biochemical characterization of cDNA clones. However, because those enzymes were isolated from transcript libraries, their genomic context, copy number, and evolutionary origin remained unknown. By generating a telomere-to-telomere genome assembly of *Salvia sclarea* and performing comparative genomic analyses across Lamiaceae, our study resolves this locus and demonstrates that the enzyme responsible for sclareol biosynthesis arose from a recent, lineage-specific tandem duplication event that is absent from closely related *Salvia* species.

Importantly, our work goes beyond identifying the duplication event by providing causal evidence linking specific amino-acid substitutions to the emergence of a new catalytic activity, through a combination of structural modeling and site-directed mutagenesis. In addition, we show that the neofunctionalized enzyme is embedded within a chromatin-regulated biosynthetic gene cluster organized into two transcriptional modules, revealing a regulatory architecture that had not been previously described for this pathway.

Thus, while the biochemical steps of sclareol biosynthesis were known, our study provides new insights into the evolutionary origin, structural basis, and regulatory organization of this metabolic innovation**,** which could not be addressed without a high-quality genome assembly and integrated genomic analyses.

Page 2, last sentence: It is unclear to what extent the findings from the present study form a basis for the sustainable production of high-value plant terpenoids. Rephrase or delete.

We thank the reviewer for this comment. We have therefore revised the sentence to clarify that our results primarily provide mechanistic insight into the evolution of terpene biosynthesis while potentially informing future engineering approaches.

"Together, our findings illustrate how enzyme innovation and regulatory rewiring can give rise to new metabolic pathways and may inform future strategies for engineering valuable plant terpenoids."

Page 6, line 22: Define "condition-dependent" and "low expression".

We thank the reviewer for this suggestion. To clarify the meaning of "condition-dependent" and "low expression", we have revised the sentence in the manuscript as follows:

Original sentence:
"Core genes exhibited broader expression across tissues and higher functional annotation, whereas specific genes showed condition-dependent or low expression, reflecting a pattern that is generic across plant pan-genomes (Fig. 2d, e)."

Revised sentence:
"Core genes exhibited broader expression across tissues and higher functional annotation, whereas species-specific genes showed low or highly restricted expression across the sampled tissues, often detectable only in particular tissues or conditions, reflecting a pattern commonly observed across plant pan-genomes (Fig. 2d, e)."

Page 6 line 26: A sentence is needed to explain the role of the cytochrome P450 superfamily in chemical diversification to a wider readership.

We thank the reviewer for this suggestion. To clarify the role of cytochrome P450 enzymes for a broader readership, we have added a sentence explaining that CYP450 monooxygenases catalyze oxidative modifications that decorate terpene scaffolds and contribute substantially to the chemical diversification of plant specialized metabolites. We have also added references supporting this statement (refs. 11 and 12):

11. Mizutani, M., Ohta, D. *Diversification of P450 genes during land plant evolution*. Annu. Rev. Plant Biol. 61, 291–315 (2010).
12. Hansen, C. C., Nelson, D. R., Møller, B. L., Werck-Reichhart, D. *Plant cytochrome P450 plasticity and evolution*. Mol. Plant 14, 1244–1265 (2021).

Page 7, line 32 and page 8, line 1: Introducing two names for one sequence is not helpful. Use either SsKSL3 and SsKSL4 or SsSCS and SsSCS-2 in the manuscript.

We thank the reviewer for this helpful suggestion. To avoid confusion, we have revised the manuscript to use a single primary gene name throughout the text. In particular, the tandem-duplicated sclareol synthases are now referred to as SsSCS and SsSCS-2.

Page 8, lines 2 and 3: Use one name for each sequence for clarity. It should be either SsCPS2.2 or SsLPPS. Revise the manuscript accordingly.

As requested by the reviewer, we have revised the manuscript to use SsLPPS as the primary gene name throughout the text. In addition, we carefully checked the entire manuscript to ensure consistent gene nomenclature across the text, figures, and supplementary materials.

Page 17, line 19: Revise "The Pfam domains of genes encoding enzymes…". Pfam refers to protein families, and domains are the structural and functional building blocks of proteins (not of genes).

We thank the reviewer for this clarification. We agree that Pfam domains refer to protein domains rather than genes. The sentence has therefore been revised as follow:

Original sentence:
"The Pfam domains of genes encoding enzymes in each catalytic step were first searched in the literature, and then, *S. sclarea* proteins containing these domains were selected as candidates."

Revised sentence:
"The Pfam domains associated with enzymes involved in each catalytic step were first identified from the literature, and *S. sclarea* proteins containing these domains were then selected as candidates."

Page 18, line 1: Specify the ACTIN gene and add the sequence as supplementary information.

The reference gene used in our study is *SsACTIN3*, which has now been explicitly specified in the Methods section. In addition, the primer sequences used for qRT–PCR amplification of *SsACTIN3* have been added to Supplementary Table 14**.**

Figures 3i, 3 j and 4d: The light blue color is barely visible in the ATAC-seq chromatin accessibility profiles.

As requested, and to improve the readability of the ATAC-seq chromatin accessibility profiles, we have adjusted the color scheme in Figures 3i, 3j, and 4d**,** replacing the light blue with a darker and more contrasting color.

Figure 4: Revise the labeling of the heat map. It is unclear what the color names refer to (e.g. midnightblue (403)).

We thank the reviewer for this comment. The labels such as "midnightblue (403)" correspond to gene modules identified by the WGCNA analysis, where module names are conventionally assigned color identifiers by the algorithm. The number in parentheses indicates the number of genes contained in the module**.** These labels therefore refer to co-expression modules rather than to the heatmap module-trait correlation color scale**.** To avoid confusion, we have clarified this point in the figure legend and slightly revised the labeling in the figure.

Page 9, lines 3-13: This paragraph seems out of place. It could be integrated into the following subsection, which reports other ATAC-seq data (see Fig. 4). The graphics in the figures 3 and 4 may need to be rearranged.

We thank the reviewer for this helpful suggestion. We agree that chromatin accessibility data are better discussed together with the analysis of transcriptional organization of the biosynthetic gene cluster. Accordingly, we reorganized the Results section so that the ATAC-seq analyses are now presented together with the WGCNA-based co-expression analysis in a single subsection describing the regulatory architecture of the cluster.

However, we retained the expression analysis of *SsLPPS* and *SsSCS* in the section describing the tandem duplication, as these data directly support the functional divergence of the duplicated enzymes. We have clarified the transitions between these sections to improve the logical flow of the Results and to avoid fragmentation between Figs. 3 and 4.

Page 10, lines 10-27: Although the authors report MeJA-induced upregulation of sclareol biosynthesis genes, the manuscript does not present corresponding measurements of sclareol accumulation. The data would provide evidence for a correlation between induced gene expression and increased sclareol production and enable more informed conclusions to be drawn.

We thank the reviewer for this insightful suggestion. We agree that measuring sclareol accumulation following MeJA treatment would provide an additional layer of evidence linking transcriptional induction to metabolite production. However, in the present study the MeJA experiment was designed primarily to assess whether genes within the diterpene biosynthetic gene cluster respond coherently to a defense-related hormonal signal, rather than to quantify changes in sclareol accumulation.

As described in the Methods section, the MeJA treatment was performed on young *S. sclarea* plantlets. While this experimental setup provides sufficient tissue for RNA extraction and qRT–PCR analysis, which require relatively small amounts of material, it does not yield enough biomass for reliable metabolomic quantification of sclareol, which typically requires substantially larger quantities of tissue.

Importantly, the objective of this experiment was to test the coordinated transcriptional response of genes within the biosynthetic gene cluster to jasmonate signaling. Our results show that the trichome-associated genes of the cluster are strongly induced by MeJA, consistent with their functional integration in a defense-related pathway and supporting the conclusion that the cluster operates as a hormonally responsive regulatory unit.

REVIEWER COMMENTS

Reviewer #1 (Remarks to the Author):

My previous comments pertained to minor inconsistencies needing clarification. The authors have satisfied these with their revised version.

Reviewer #2 (Remarks to the Author):

The authors have provided clear and thorough responses to all issues raised in the previous round of review, and have made comprehensive and well-structured revisions, effectively addressing the prior shortcomings. The paper is recommended for acceptance without further modifications.

Reviewer #3 (Remarks to the Author):

The revisions made have significantly improved the manuscript's clarity and scientific rigor. My questions and comments have been addressed.
